# Discovery of candidate tumor biomarkers for treatment with intraperitoneal chemotherapy for ovarian cancer

**DOI:** 10.1038/srep21591

**Published:** 2016-02-17

**Authors:** Brandon-Luke L. Seagle, Kevin H. Eng, Judy Y. Yeh, Monica Dandapani, Emily Schiller, Robert Samuelson, Kunle Odunsi, Shohreh Shahabi

**Affiliations:** 1 Department of Obstetrics, Gynecology and Reproductive Sciences, Western Connecticut Health Network, Danbury, CT, U.S.A; 2 Department of Biostatistics and Bioinformatics, Roswell Park Cancer Institute, Buffalo, NY, U.S.A; 3 Department of Gynecologic Oncology, Roswell Park Cancer Institute, Buffalo, NY, U.S.A; 4 Division of Gynecologic Oncology, Department of Obstetrics and Gynecology, Prentice Women’s Hospital, Northwestern University Feinberg School of Medicine, Chicago, IL, U.S.A

## Abstract

Tumor mRNA expression was used to discover genes associated with worse survival or no survival benefit after intraperitoneal (IP) chemotherapy. Data for high grade serous ovarian cancer patients treated with IP (n = 90) or IV-only (n = 398) chemotherapy was obtained from The Cancer Genome Atlas. Progression free survival (PFS) and overall survival (OS) were compared between IP and IV groups using Kaplan-Meier analysis and Cox regression. Validations were performed by analyses of microarray and RNA-Seq mRNA expression data. PFS and OS were compared between IP and IV groups by permutation testing stratified by gene expression. P-values are two-tailed. IP chemotherapy increased PFS (26.7 vs 16.0 months, HR 0.43 (0.28–0.66), p = 0.0001) and OS (49.6 vs 38.2 months, HR 0.46 (0.25–0.83), p = 0.01). Increased expression of *NCAM2* and *TSHR* and decreased expression of *GCNT1* was associated with decreased PFS and OS after IV chemotherapy (p < 0.05). High tumor expression of *LMAN2*, *FZD4*, *FZD5*, or *STT3A* was associated with no significant PFS increase after IP compared to IV chemotherapy. Low expression of *APC2* and high expression of *FUT9* was associated with 5.5 and 7.2 months, respectively, decreased OS after IP compared to IV chemotherapy (p ≤ 0.007).

Ovarian cancer is the deadliest gynecologic malignancy, with 44.6% five-year overall survival (OS) and 1% lifetime mortality risk^1^. Standard management includes surgical staging and cytoreduction followed by adjuvant intravenous (IV) platinum-taxane chemotherapy^2^. Compared to treatment with only IV chemotherapy, addition of adjuvant intraperitoneal (IP) chemotherapy increased progression free survival (PFS) and OS among patients with stage III ovarian cancer^3^,^4^,^5^. Gynecologic Oncology Group protocol 172 reported a median PFS and OS increase of 5.5 and 15.9 months, respectively, after IP chemotherapy^5^. A recent systemic analysis confirmed increased PFS and OS after adjuvant IP chemotherapy^6^. Survival advantage after adjuvant IP chemotherapy extended beyond 10 years^7^.

Despite available strategies for preemptive management of side effects of IP chemotherapy, widespread adoption of IP chemotherapy remains limited by toxicities^8^,^9^. Prognostic factors including age, histology, and cytoreduction aided selection of patients for IP chemotherapy^10^. Low *BRCA1* protein expression measured by immunohistochemistry of primary tumor specimens was associated with 36 months increased OS among patients treated with IP compared to IV-only adjuvant chemotherapy^11^. In an effort to identify biomarkers of response to IP chemotherapy among high grade serous ovarian cancer (HGS OvCa) patients, we used data from The Cancer Genome Atlas (TCGA) to discover genes with mRNA expression levels that were associated with PFS and OS after treatment with adjuvant IP or IV chemotherapy.

## Methods

### Case selection and data collection

Chemotherapy information was from the TCGA drug dataset^12^. Patients were included in the study based on their chemotherapy exposures. Patients who received adjuvant IP ± IV (n = 90) or only IV (n = 398) chemotherapy were included (Supplementary Dataset 1). Patients who received neoadjuvant chemotherapy (n = 1) or who had no available clinical outcome data (n = 5) were excluded. We used Level 3 mRNA expression data from primary tumor specimens measured by Affymetrix U133A microarray, annotated with the hthgu133a.db package^13^,^14^, or by RNA sequencing (RNA-Seq). TCGA previously described specimen collection and assay validation^12^,^13^. Clinical and mRNA expression data was downloaded using CGDS-R^15^,^16^. Data was public per TCGA polices^17^.

### Survival analysis by chemotherapy exposure

We used the R platform for statistical computing^18^,^19^. P-values were two-tailed. Demographic and outcome information were compared using statistical tests indicated with Results. Incomplete clinical data reporting to TCGA was common. Cases with missing data were excluded from statistical comparisons. Cytoreduction was considered optimal if residual disease was ≤10 mm after surgery. PFS and OS data was truncated at 60 months to prevent biasing results by long surviving individuals and to match a restriction time of 60 months set for restricted mean survival (RMS) calculations. Empiric Kaplan-Meier (KM) survival analysis and Cox proportional-hazards regression adjusted for covariates age, surgical stage, histologic grade, cytoreduction status and race were used to compare PFS and OS by chemotherapy route (IP versus IV). For KM analysis of all cases (n = 488), 96 and 42 cases were omitted due to missing OS and PFS data, respectively. For KM analysis of optimally cytoreduced cases (n = 282), 47 and 18 cases were omitted due to missing OS and PFS data, respectively. For multivariate Cox regression of OS and PFS for all cases (n = 488), 178 and 144 cases, respectively, were omitted due to missing data. For multivariate Cox regression of OS and PFS for optimally cytoreduced cases (n = 282), 52 and 22 cases, respectively, were omitted due to missing data.

### Discovery and validation of differentially expressed genes

Within each adjuvant chemotherapy group, cases were stratified by PFS time <or ≥12 months. Twelve months was used because cancers that recurred or progressed before 12 months were likely chemoresistant. Patients who undergo surgical staging and six cycles of adjuvant chemotherapy often reach 6 months after completion of chemotherapy, the time at which platinum sensitivity is designated, about 12 months after surgery. A diagram of the exploratory gene analysis and validation steps is shown in Supplementary Table 1. A t-test compared microarray mRNA expression between PFS strata to discover differentially expressed genes. Standard t-testing was used because the results are fully reproducible unlike permutation methods of microarray data analysis, which yield a larger number of differentially expressed genes with a higher number of false positive discoveries. Microarray data was used for exploratory analysis because too few patients had RNA-Seq data for gene discovery. Genes evaluated were listed under KEGG pathways identified by the microarray annotation^14^,^20^. Multiple comparison FDR adjusted p-values and fold changes were calculated for differentially expressed genes. As a first validation that differentially expressed genes are associated with differences in survival, Cox regression of PFS and OS by microarray mRNA expression with inclusion of covariates age, surgical stage and histologic grade was performed for all differentially expressed genes. For multivariate Cox regression (microarray) of OS and PFS for IP patients (n = 90), 30 and 18 cases, respectively, were omitted due to missing data. For multivariate Cox regression (microarray) of OS and PFS for IV patients (n = 398), 121 and 90 cases, respectively, were omitted due to missing data. As a second validation, multivariate Cox regression for differentially expressed genes was performed with the RNA-Seq data. For multivariate Cox regression (RNA-Seq) of OS and PFS for IP patients (n = 34), 7 and 2 cases, respectively, were omitted due to missing data. For multivariate Cox regression (RNA-Seq) of OS and PFS for IV patients (n = 187), 34 and 21 cases, respectively, were omitted due to missing data. Genes significantly associated with OS and/or PFS by both validations were considered positive discoveries, creating a stringent validation.

### Survival differences between IP versus IV chemotherapy groups subdivided by gene expression

RMS curves were plotted for differentially expressed genes as functions of normalized relative microarray mRNA expression^21^. RMS times were the areas under the Cox regression adjusted survival curves of OS and PFS using a restriction time of 60 months^21^,^22^. For univariate Cox regression of OS and PFS for IP patients (n = 90), 16 and 2 cases, respectively, were omitted due to missing data. For univariate Cox regression of OS and PFS for IV patients (n = 398), 80 and 40 cases, respectively, were omitted due to missing data. Univariate Cox regression was used to calculate RMS times for plotting RMS curves. The p-values for OS and PFS reported on RMS curves in Results are multivariate regression derived p-values after adjustment for age, stage, and grade. Tumor mRNA expression was normalized by ordering TCGA expression values from least to greatest and calculating a continuous relative quantile value from 0 to 1 for each expression value. Normalized relative expression values were used to place cases along the x-axis of RMS curves. RMS curves represent the mean survival times (y-axis values) of patients with up to 60 months clinical follow-up given a particular relative mRNA expression level (the x-axis values). Permutation testing (10,000 permutations) was done to test the significance of the difference in mean OS and PFS between patients treated with IP versus IV chemotherapy given a specified range of gene expression. The null hypothesis was that patients treated with IP chemotherapy have significantly increased OS and PFS at all expression levels of each gene. This null hypothesis is consistent with survival analysis of the clinical data (see Results). Findings from permutation testing with microarray expression data were validated by identical permutation testing using RNA-Seq data. Any gene for which the null hypothesis is rejected by both methods is considered a candidate biomarker that warrants experimental validation.

## Results

### Patient and chemotherapy information

Table 1 shows patient and disease characteristics for the adjuvant IP and IV chemotherapy groups. Patients who received IP chemotherapy were younger (55.3 versus 59.9 years, p < 0.001) and more often achieved optimal cytoreduction (86.6% versus 70.4%, p = 0.007). Adjuvant chemotherapy administration for the IP and IV groups is shown in Table 2. In the IP group, 89/90 (99%) patients received both IP and IV chemotherapy. Median IV chemotherapy cycles completed was 6 in both groups. In the IP group, 51% of patients completed 6 or more cycles of IP chemotherapy.

### Survival outcomes by chemotherapy route

**Figure 1:**
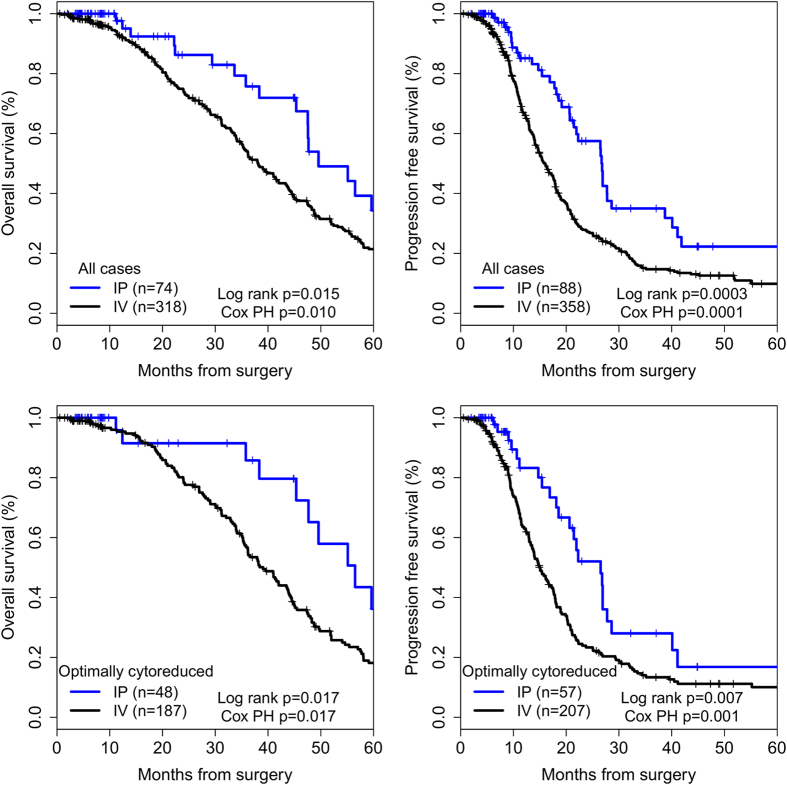
Kaplan-Meier survival curves for patients treated with intraperitoneal (IP) and intravenous (IV) chemotherapy. Log rank is log rank p-value from Kaplan-Meier analysis. Cox PH is multivariate Cox proportional hazards regression p-value for chemotherapy route with adjustment for age, surgical stage, histologic grade, cytoreduction status, and race.

KM survival curves comparing survival by chemotherapy route are shown in Fig. 1. IP chemotherapy was associated with increased PFS (26.7 versus 16.0 months, HR (95% CI) 0.43 (0.28–0.66), p = 0.0001) and OS (49.6 versus 38.2 months, HR (95% CI) 0.46 (0.25–0.83), p = 0.01) among all (n = 488) cases. Analyzing optimally cytoreduced cases (n = 281) separately, IP chemotherapy was associated with increased PFS (26.5 versus 15.1 months, HR (95% CI) 0.46 (0.29–0.73), p = 0.001) and OS (56.5 versus 39.1 months, HR (95% CI) 0.42 (0.21–0.85), p = 0.017). Age, stage, grade, cytoreduction status, and race were not significant covariates. There were no significant differences in OS or PFS among suboptimally cytoreduced patients (Supplementary Figure 1).

### Gene expression analysis by PFS stratification

Supplementary Table 2 shows patient and disease characteristics for chemotherapy groups stratified by PFS < or ≥12 months. Only 9/90 (10%) patients in the IP group had PFS < 12 months. The odds ratio for PFS < 12 months in the IP versus IV group was 0.38, p = 0.011 (Fisher’s Exact test). Thirty-six and three genes were differentially expressed between PFS strata within the IV and IP groups, respectively (Supplementary Dataset 2 and Supplementary Table 3). Differentially expressed genes that were significantly associated with OS and/or PFS by multivariate regression are tabulated with adjusted (FDR) p-values and fold changes for differential expression between PFS strata, as well as with HRs for the associations of microarray or RNA-Seq mRNA expression with PFS and OS (Table 3).

### Associations of gene expression with route-specific chemoresistance

Univariate and multivariate associations of microarray mRNA expression with PFS and OS were used to validate differentially expressed genes (Validation step 1) (Supplementary Tables 3 and 4). Hazards ratios (HRs) of genes significantly associated with OS and/or PFS ranged from 0.39 to 6.35 (Supplementary Table 4). To make HRs comparable, gene expression values were scaled such that adjusted HRs are interpretable as the change in hazard per 1 standard deviation change in gene expression (Table 3). For instance, an adjusted HR of 1.30 (example: *ASPA*, overall survival) indicates that the risk of death increased 30% for each 1 standard deviation increase in microarray mRNA expression across the range of observed expression values. Since expression data varies over several standard deviations for each gene, survival effects for some genes are large comparing the lower and upper limits of gene expression (see Supplementary RMS curves for visual illustrations). Almost half (19/39, 49%) of the differentially expressed genes were associated with PFS and/or OS by multivariate regression using microarray data (Validation step 1) (Table 3). Only 5/39 (12.8%) of the differentially expressed genes were associated with PFS and/or OS by multivariate regression using RNA-Seq data (Validation step 2) (Supplementary Tables 5 and 3). Increased expression of *NCAM2* and *TSHR* and decreased expression of *GCNT1* was associated with decreased PFS and OS after IV chemotherapy (p < 0.05) (Table 3).

### Candidate biomarker discovery for patient selection for IP chemotherapy

**Figure 2:**
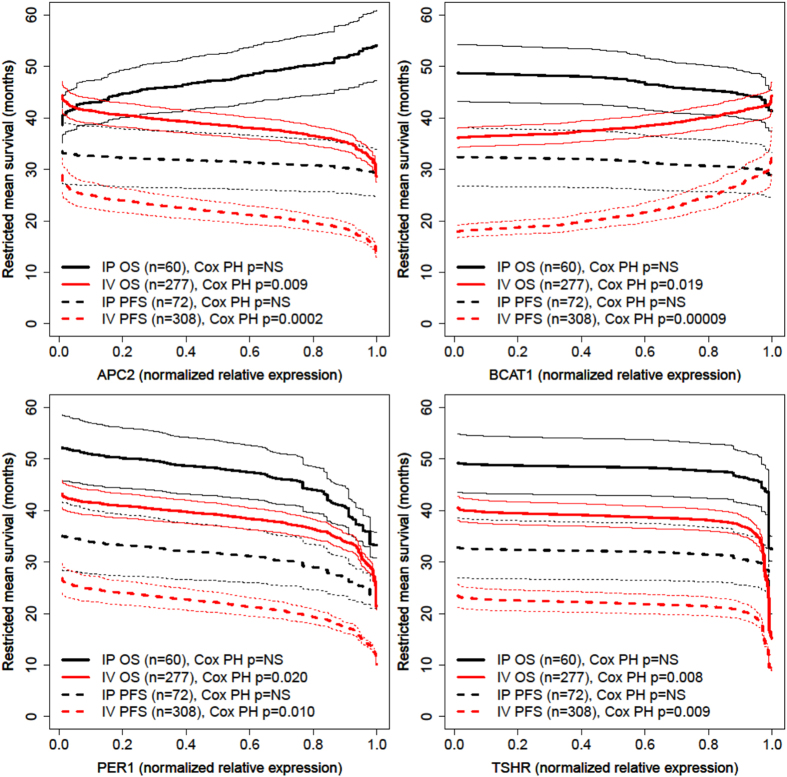
Restricted mean survival curves by normalized relative gene expression, shown with pointwise 95% confidence intervals. IP: Intraperitoneal. IV: Intravenous. PFS: Progression free survival. OS: Overall survival. Cox PH: multivariate Cox proportional hazards regression p-value for the association of gene expression with indicated survival times after adjustment for age, surgical stage and histologic grade.

Since patients treated with IP chemotherapy typically have significantly increased OS and PFS compared to patients who receive IV-only chemotherapy, biomarker evaluation for response to IP chemotherapy may aim to discover primary tumor biomarkers that are associated with worse or no better survival after IP chemotherapy. RMS curves demonstrate PFS and OS by relative gene mRNA expression (Supplementary RMS curves) allowing for evaluation of survival as a function of gene expression across the range of observed gene expression values. Pointwise 95% confidence intervals around each univariate RMS curve allow visual comparison of OS and PFS between IP and IV groups (Supplementary RMS curves). Illustrative RMS curves are shown (Fig. 2). Comparing univariate RMS curves and multivariate RMS times between IP and IV groups evaluates differentially expressed genes as biomarkers for treatment benefit from IP chemotherapy. For instance, according to microarray data, patients whose tumors had low expression of *APC2* or high expression of *BCAT1* experienced less survival benefit from the addition of IP chemotherapy to their treatment (Fig. 2). Patients whose tumors had high expression of *PER1* or *TSHR* experienced decreased survival compared to patients whose tumors did not have high expression of these genes, with the associations of gene expression and survival outcomes reaching statistical significance in the larger cohort of patients who received IV-only adjuvant chemotherapy (Fig. 2). Table 4 lists differentially expressed genes for which there is evidence to reject the null hypothesis based on microarray data and designate the gene as a candidate biomarker for patient selection for IP chemotherapy (see Methods above). For example, patients with high expression (upper 10^th^ percentile) of *FZD5* showed an expected overall survival benefit among the IP chemotherapy group but did not show a significant PFS benefit (no significant difference between IP and IV-only groups) (Table 4). Table 4 also shows the results of validation permutation testing using RNA-Seq data. High tumor expression of *LMAN2*, *FZD4*, *FZD5*, or *STT3A* was associated with no significant PFS increase after IP compared to IV-only chemotherapy. Low expression of *APC2* and high expression of *FUT9* was associated with 5.5 and 7.2 months, respectively, decreased OS after IP compared to IV-only chemotherapy (p ≤ 0.007).

## Discussion

Adjuvant IP chemotherapy is associated with increased PFS and OS in TCGA data. A large and increasing number of publications reported survival outcomes of TCGA ovarian cancer patients without adjusting for chemotherapy route as a potential confounder. Reanalysis of previously reported survival outcomes to adjust for chemotherapy route as a potential confounder may be needed given that IP chemotherapy use was common among TCGA HGS OvCa patients and that receiving IP chemotherapy was associated with large and highly significant increases in PFS and OS.

A detailed review of the tumor biology of all candidate biomarkers is beyond the scope of this report. DAVID gene functional classification failed to reveal any clusters among differentially expressed genes^23^. Three differentially expressed genes, *APC2*, *FZD4*, and *FZD5*, which had mRNA levels that were significantly associated with OS and PFS, are members of the Wnt signaling pathway. The observation that increased expression of *APC2* and decreased expression of *FZD4* and *FZD5* was associated with decreased OS and PFS among patients treated with IV chemotherapy suggests that downregulation of Wnt signaling may play a role in chemoresistance^24^,^25^. The Wnt pathway may be targeted in cancer trials^25^,^26^. Recently, advanced ovarian cancers with increased membrane β-catenin expression by immunohistochemistry were shown to have decreased PFS and increased platinum-resistance, consistent with our findings^27^.

Limitations of our study include incomplete clinical data reporting to TCGA, a relatively small number of patients who received IP chemotherapy, and also small numbers of tumors that were analyzed by RNA-Seq, especially among the IP group. During the RNA-Seq validation, many survival associations trended (p = 0.05–0.10) but failed to reach significance due to few cases having RNA-Seq data. Clinical and chemotherapy information reported to TCGA was also not verified for correctness. We could not perform validation in another patient cohort because no other suitable data is available. To our knowledge, TCGA provides the only cohort of ovarian cancer patients treated with IP chemotherapy for which there is currently transcriptome or proteome data. Also, tumor protein expression was not confirmed experimentally by immunohistochemistry of primary tumor specimens. The TCGA reverse phase protein array does not include any of the differentially expressed genes and therefore the TCGA proteomics data could not be used as a potential protein-level validation of our transcription-level findings. In addition, the process of gene discovery by t-testing and gene validation with limited RNA-Seq data is very stringent. Thus, there are likely additional genes that are significantly associated with survival outcomes among this patient cohort. Targeted rather than exploratory analyses of well-known molecular pathways driving cancer development or chemotherapy resistance may lead to discovery of additional genes with similar survival associations after adjusting for chemotherapy exposure.

Strengths of our study include use of a standard and fully reproducible statistical test (the t-test) to compare gene expression levels between groups. We also analyzed gene mRNA expression levels as a continuous variable in all regression models of PFS or OS. Dichotomizing or otherwise arbitrarily subdividing cases by gene expression thresholds is very commonly reported in the literature but is less informative than RMS analysis. RMS curves illustrate the relationships of gene expression and survival. We provide TCGA case identifiers with chemotherapy route to ease reproduction of our findings and reanalysis of previous studies.

Adjuvant IP chemotherapy of TCGA patients was associated with increased PFS and OS similar to randomized trials^3^,^4^,^5^. Our study is the second study, after one other recent report, to demonstrate off-trial survival advantage associated with adjuvant IP chemotherapy use that included some common “modified regimens” that are often individual provider-designed to decrease toxicity^28^. Our study reports exploration of transcriptome data motivated by a practical, clinically-oriented question: Given the increased morbidity of IP chemotherapy, are there biomarkers for response to IP chemotherapy that may aid selection of patients for IP chemotherapy? We discovered candidate biomarkers that are significantly associated with chemoresistance and decreased survival, or lack of benefit from IP chemotherapy. Our findings generate hypotheses regarding route-specific chemoresistance that may be tested using *in vivo* models. A targeted clinical assay for efficient measurement of primary tumor gene expression of these candidate biomarkers may be developed. Some gene products may be amenable to measurement in serum or ascites, or by primary tumor immunohistochemistry. Our findings may be corroborated at the level of protein expression by performing immunohistochemical analysis of primary tumor specimens, if made available, from a previous randomized trial of IP versus IV chemotherapy^3^,^4^,^5^. If similar results are obtained, a study of specimens from a previous randomized trial may efficiently provide validation of these candidate biomarkers as clinical biomarkers for patient selection for IP chemotherapy.

## Additional Information

**How to cite this article**: Seagle, B.-L. L. *et al.* Discovery of candidate tumor biomarkers for treatment with intraperitoneal chemotherapy for ovarian cancer. *Sci. Rep.* **6**, 21591; doi: 10.1038/srep21591 (2016).

## Supplementary Material

Supplementary Information

Supplementary Dataset 1

Supplementary Dataset 2

## Figures and Tables

**Table 1:** Comparison of patient and case characteristics between adjuvant chemotherapy groups.

	IP group (n = 90)	IV group(n = 398)	p-value
*Mean age*	55.3	59.9	<0.001^a^
*Stage*			0.997^b^
IIA/B/C	1	21	
IIIA/B	4	23	
IIIC	63	250	
IV	9	58	
*Grade*			0.693^b^
G2	8	43	
G3	66	302	
*Race*			0.970^c^
Asian	2	13	
Black	4	17	
White	81	348	
Other	0	3	
*Cytoreduction*			0.006^c^
Optimal	58	224	
Suboptimal	9	94	
*Adjuvant Chemotherapy Outcome*			0.007^c^
Complete Response	58	210	
Partial Response	3	51	
Progressive Disease	2	29	
Stable Disease	4	18	
*Platinum Status*			0.037^c^
Resistant	8	80	
Sensitive	37	159	

Numbers shown for each comparison do not add to the total number of patients in each adjuvant chemotherapy group due to incomplete data reporting or inadequate follow up time. Missing data is omitted from statistical comparisons. IP: Intraperitoneal. IV: Intravenous.

^a^T-test.

^b^Kruskal-Wallis test.

^c^Fisher’s Exact test.

**Table 2:** Adjuvant chemotherapy administration.

Study group	IP group (n = 90)		IV group(n = 398)
Route of administration	IP	IV	IV
Days to start(Median ± SD)	42 ± 83	26 ± 16	26 ± 21
Cycles given(Median ± SD)	6 ± 1.9	6 ± 1.4	6 ± 1.7
Cycles completed	n (%)	n (%)	n (%)
0 cycles	0 (0%)	1 (1%)	0 (0%)
1–2 cycles	12 (13%)	0 (0%)	9 (2%)
3–5 cycles	30 (33%)	1 (1%)	35 (9%)
6+ cycles	46 (51%)	88 (98%)	334 (84%)
Not reported	2 (2%)	0 (0%)	20 (5%)
*Single Agent*	*27 (30%)*	*0 (0%)*	*12 (3%)*
Cisplatin	22 (24%)	0 (0%)	0 (0%)
Carboplatin	2 (2%)	0 (0%)	12 (3%)
Paclitaxel	2 (2%)	0 (0%)	0 (0%)
Doxorubicin	1 (1%)	0 (0%)	0 (0%)
*Multiple Agent*	*63 (70%)*	*89 (99%)*	*386 (97%)*
Cisplatin	47 (52%)	10 (12%)	34 (9%)
Carboplatin	23 (26%)	79 (88%)	354 (89%)
Paclitaxel	56 (62%)	89 (99%)	350 (88%)
Docetaxel	11 (12%)	14 (15%)^a^	36 (9%)
Gemcitabine	2 (2%)	3 (4%)	25 (6%)
Doxorubicin	0 (0%)	0 (0%)	11 (3%)
Bevacizumab	1 (1%)	0 (0%)	6 (2%)
Cyclophosphamide	1 (1%)	0 (0%)	10 (3%)
Topotecan	1 (0%)	0 (0%)	8 (2%)

IP: Intraperitoneal. IV: Intravenous. SD: Standard deviation.

^a^15% of patients may have switched from paclitaxel to docetaxel during adjuvant treatment.

**Table 3:** Associations of differentially expressed gene tumor mRNA expression levels with survival outcomes.

Group	Gene	p^a^	Fold change^b^	Microarray mRNA expression		RNASeq mRNA expression	
				OS	PFS	OS	PFS
				HR (95% CI)^c^, p	HR (95% CI)^c^, p	HR (95% CI)^c^, p	HR (95% CI)^c^, p
IV	FZD5	0.027	1.285	**0.75 (0.64**–**0.88), <0.001**	**0.73 (0.64**–**0.84), <0.001**	0.91 (0.69–1.20), 0.513	0.94 (0.72–1.21), 0.618
IV	BCAT1	0.006	1.621	**0.83 (0.71**–**0.97), 0.019**	**0.76 (0.66–0.87), <0.001**	0.95 (0.80–1.14), 0.591	1.02 (0.82–1.25), 0.893
IV	GCNT1	0.001	1.189	0.89 (0.76–1.05), 0.173	**0.79 (0.68–0.91), 0.001**	**0.76 (0.59–0.97), 0.031**	**0.79 (0.66–0.95), 0.011**
IV	FZD4	0.027	1.291	**0.86 (0.74–1.00), 0.044**	**0.81 (0.70–0.93), 0.002**	0.85 (0.66–1.09), 0.188	0.82 (0.65–1.03), 0.087
IV	PYCR1	0.046	1.170	0.98 (0.85–1.13), 0.749	**0.82 (0.72-.93), 0.002**	1.04 (0.85–1.29), 0.685	0.88 (0.73–1.07), 0.196
IV	HLCS	0.034	1.043	**0.82 (0.70–0.95), 0.010**	**0.82 (0.72–0.94), 0.006**	0.85 (0.70–1.03), 0.098	0.98 (0.83–1.16), 0.843
IV	NANS	0.006	1.222	1.01 (0.86–1.17), 0.948	**0.84 (0.73–0.96), 0.012**	1.04 (0.86–1.25), 0.722	0.87 (0.73–1.04), 0.120
IV	STT3A	0.017	1.220	0.87 (0.75–1.01), 0.066	**0.86 (0.75–0.97), 0.016**	0.92 (0.74–1.13), 0.432	0.84 (0.70–1.01), 0.067
IV	PLA2G2F	0.019	0.962	1.08 (0.92–1.28), 0.337	1.10 (0.95–1.27), 0.216	**1.34 (1.10–1.64), 0.004**	**1.64 (1.04–2.60), 0.033**
IV	PAH	0.007	0.956	**1.24 (1.07–1.44), 0.005**	1.12 (0.97–1.29), 0.118	1.08 (0.88–1.32), 0.473	1.09 (0.90–1.32), 0.383
IV	GCNT4	0.046	0.967	1.12 (0.95–1.31), 0.167	**1.15 (1.00–1.32), 0.047**	0.52 (0.23–1.15), 0.104	0.80 (0.64–1.01), 0.056
IV	CMA1	0.018	0.961	1.06 (0.93–1.20), 0.370	**1.15 (1.02–1.30), 0.024**	1.07 (0.92–1.24), 0.379	1.11 (0.96–1.30), 0.139
IV	RPE65	0.016	0.971	1.04 (0.90–1.20), 0.565	**1.16 (1.02–1.31), 0.021**	0.95 (0.47–1.94), 0.890	1.21 (0.77–1.90), 0.411
IV	NCAM2	0.041	0.960	**1.19 (1.03–1.38), 0.020**	**1.17 (1.03–1.34), 0.018**	**1.21 (1.02–1.42), 0.025**	**1.23 (1.05–1.45), 0.011**
IV	TSHR	0.018	0.879	**1.21 (1.05–1.40), 0.008**	**1.17 (1.04–1.33), 0.009**	**1.71 (1.31–2.22), <0.001**	**1.64 (1.22–2.21), 0.001**
IV	THPO	0.002	0.967	**1.16 (1.01–1.35), 0.041**	**1.18 (1.03–1.35), 0.018**	1.11 (0.80–1.55), 0.533	0.84 (0.66–1.05), 0.121
IV	ASPA	0.010	0.950	**1.30 (1.13–1.51), <0.001**	**1.19 (1.04–1.36), 0.011**	1.03 (0.86–1.24), 0.736	1.06 (0.87–1.29), 0.560
IV	PER1	0.003	0.877	**1.21 (1.03–1.41), 0.020**	**1.21 (1.05–1.40), 0.010**	1.01 (0.78–1.29), 0.955	1.15 (0.94–1.41), 0.170
IV	APC2	0.003	0.956	**1.22 (1.05–1.42), 0.009**	**1.28 (1.12–1.45), <0.001**	0.91 (0.74–1.12), 0.360	1.01 (0.88–1.16), 0.897
IV	FUT9	0.049	0.971	1.08 (0.94–1.24), 0.271	**1.28 (1.14–1.43), <0.001**	0.99 (0.81–1.22), 0.949	1.11 (0.92–1.32), 0.269
IV	AASS	0.010	0.922	0.98 (0.65–1.50), 0.945	1.31 (0.92–1.86), 0.134	0.98 (0.82–1.17), 0.823	**1.18 (1.00–1.38), 0.044**

OS: Overall survival; PFS: Progression free survival.

^a^Adjusted p-values for differentially expressed genes within each adjuvant chemotherapy group after stratification by progression free survival (PFS) < versus ≥12 months.

^b^Fold change indicates relative level of tumor mRNA expression among patients with PFS ≥12 months compared to patients with PFS < 12 months.

^c^HR (95% CI): Hazard ratio (95% Confidence Interval), p-value calculated by Cox proportional hazards regression adjusted for covariates age, surgical stage and histologic grade. HRs are adjusted to 1 standard deviation of gene expression for each gene to aid between-gene interpretations regarding the association of gene expression with survival. For instance, a HR of 1.30 for gene X would indicate that the risk of death increased 30% for each 1 standard deviation increase in mRNA expression of gene X across the range of observed expression values of gene X. Significant associations are in bold type for convenient reference.

**Table 4:** Comparisons of mean RMS times between patients treated with IP versus IV chemotherapy selected by relative expression quantile range.

Gene	Expression (quantile range)	Microarray mRNA expression				RNA-Seq mRNA expression			
		∆PFS_IP-IV_(months)	p-value^a^	∆OS_IP-IV_(months)	p-value^a^	∆PFS_IP-IV_(months)	p-value^a^	∆OS_IP-IV_(months)	p-value^a^
DGAT1	0.9–1.0	−5.8	<0.0001	10.4	<0.0001	5.3	0.0021	23.4	<0.0001
BCAT1	0.9–1.0	−4.3	0.0014	5.9	<0.0001	7.8	<0.0001	15.2	<0.0001
GPAA1	0.9–1.0	−2.1	0.1645	−0.2	0.8921	8.0	0.0004	18.7	<0.0001
TSHR	0.9–1.0	−0.4	0.5353	0.8	0.5344	45.9	<0.0001	21.4	<0.0001
LMAN2	0.9–1.0	−**0.3**	**0.8279**	7.0	<0.0001	**2.3**	**0.2984**	0.3	0.307
FZD5	0.9–1.0	−**0.1**	**0.9550**	9.1	<0.0001	−**8.7**	<**0.0001**	14.1	<0.0001
FZD4	0.9–1.0	**1.1**	**0.4258**	7.3	<0.0001	−**1.5**	**0.4229**	12.8	<0.0001
STT3A	0.9–1.0	**1.4**	**0.4101**	3.4	0.0093	−**6.2**	**0.0046**	−2.3	0.1323
CD82	0.0–0.1	4.8	0.0007	−10.4	<0.0001	8.5	0.0247	16.4	0.0249
ST6GAL1	0.9–1.0	5.1	0.0003	−0.1	0.9499	−11.9	<0.0001	12.7	<0.0001
APC2	0.0–0.1	8.6	<0.0001	−**5.5**	**0.0007**	2.9	0.3207	−**18.9**	**0.094**
SUCLG2	0.0–0.1	9.1	<0.0001	0.6	0.6370	6.5	0.0351	7.1	<0.0001
UGDH	0.0–0.1	10.9	<0.0001	0.4	0.7292	15.9	<0.0001	5.1	0.0708
MTHFR	0.0–0.1	11.8	<0.0001	2.7	0.0972	2.9	0.1913	19.4	0.0009
FUT9	0.9–1.0	12.9	<0.0001	−**7.2**	<**0.0001**	−1.9	0.1752	−**17.9**	<**0.0001**

RMS: Restricted mean survival. ∆PFS_IP-IV_: Mean RMS time for progression free survival of patients from the IP group minus mean RMS time for progression free survival of patients from the IV group, with patients selected by the indicated relative expression quantile range. ∆OS_IP-IV_: Mean RMS time for overall survival of patients from the IP group minus mean RMS time for overall survival of patients from the IV group, with patients selected by the indicated relative expression quantile range. Quantile range 0.90–1.00 refers to the top 10% of all cases from the IP and IV groups with the highest expression of the indicated gene. RMS times calculated from multivariate Cox proportional hazards regression including covariates age, surgical stage, and histologic grade.

^a^Two-tailed p-value from permutation test of 10,000 permutations. Bold type shows survival differences where survival of patients treated with IP chemotherapy was either significantly decreased or not significantly different compared to patients treated with IV-only chemotherapy, as suggested by separate analyses of the microarray and RNA-Seq expression data.
