# Supplementary material for: Discovery of candidate tumor biomarkers for treatment with intraperitoneal chemotherapy for ovarian cancer
Source: Sci Rep. 2016 Feb 17;6:21591. doi: 10.1038/srep21591 (PMC4756718; doi:10.1038/srep21591)
Supplement: Supplementary Information [file srep21591-s1.pdf]

Discovery of candidate tumor biomarkers for treatment with intraperitoneal chemotherapy for ovarian cancer

Brandon-Luke L. Seagle[1], Kevin H. Eng[2], Judy Y. Yeh[1], Monica Dandapani[1], Emily Schiller[2], Robert Samuelson[1], Kunle Odunsi[3], and Shohreh Shahabi[4,*]

1. Department of Obstetrics, Gynecology and Reproductive Sciences, Western Connecticut Health Network, Danbury, CT
2. Department of Biostatistics and Bioinformatics, Roswell Park Cancer Institute, Buffalo, NY
3. Department of Gynecologic Oncology, Roswell Park Cancer Institute, Buffalo, NY
4. Division of Gynecologic Oncology, Department of Obstetrics and Gynecology, Prentice Women′s Hospital, Northwestern University Feinberg School of Medicine, Chicago, IL

Supplementary Information

Supplementary RMS curves. Restricted mean survival curves by normalized relative gene expression, shown with pointwise 95% confidence intervals, for all differentially expressed genes. IP: Intraperitoneal. IV: Intravenous. PFS: Progression free survival. OS: Overall survival. Cox PH: multivariate Cox proportional hazards regression p-value for the association of gene expression with indicated survival times after adjustment for age, surgical stage and histologic grade.

Supplementary Figure 1. Kaplan-Meier survival curves for suboptimally cytoreduced patients by route of adjuvant chemotherapy administration.

Supplementary Table 1. Diagram of exploratory gene analysis and validation analyses performed by this study.

Supplementary Table 2. Adjuvant chemotherapy groups compared by progression free survival times < 12 months versus ≥ 12 months.

Supplementary Table 3. Associations of differentially expressed gene tumor microarray mRNA expression levels with survival outcomes (univariate analysis).

Supplementary Table 4. Associations of differentially expressed gene tumor microarray mRNA expression levels with survival outcomes (multivariate analysis).

Supplementary Table 5. Associations of differentially expressed gene tumor RNA-Seq mRNA expression levels with survival outcomes (multivariate analysis).

Supplementary Dataset 1. TCGA case identification codes organized by chemotherapy route (separate Excel file).

Supplementary Dataset 2. DAVID analysis report of differentially expressed genes (separate Excel file).

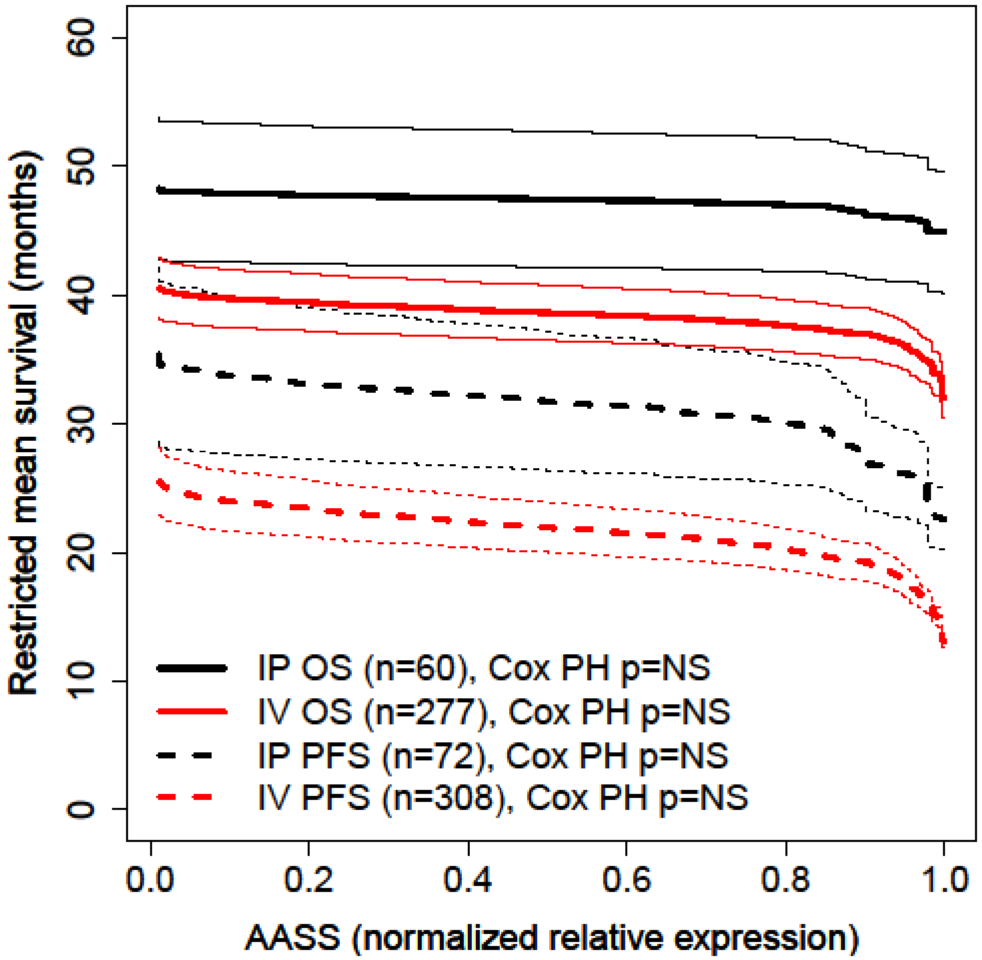

Restricted mean survival (months)
60
50
40
30
20
10
0
0.0
0.2
0.4
0.6
0.8
1.0
AASS (normalized relative expression)
IP OS (n=60), Cox PH p=NS
IV OS (n=277), Cox PH p=NS
IP PFS (n=72), Cox PH p=NS
IV PFS (n=308), Cox PH p=NS

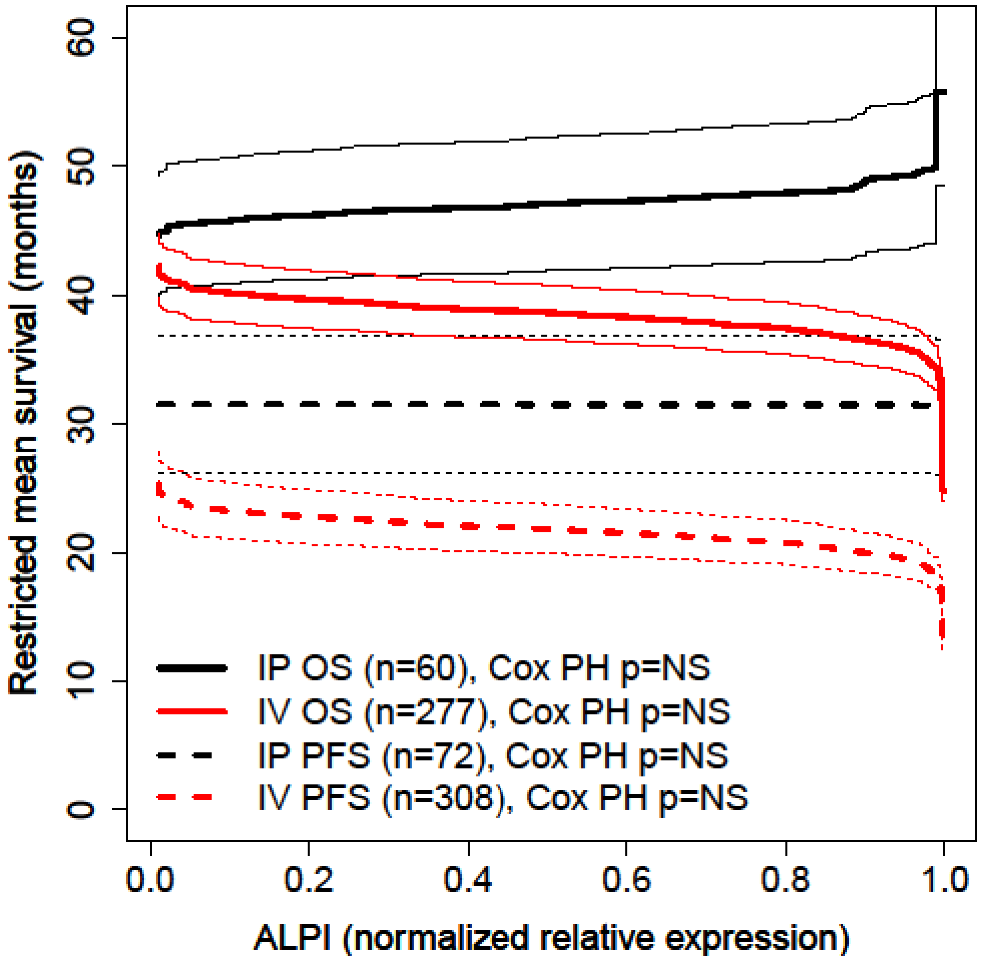
Restricted mean survival (months)
0
10
20
30
40
50
60
0.0
0.2
0.4
0.6
0.8
1.0
ALPI (normalized relative expression)
IP OS (n=60), Cox PH p=NS
IV OS (n=277), Cox PH p=NS
IP PFS (n=72), Cox PH p=NS
IV PFS (n=308), Cox PH p=NS

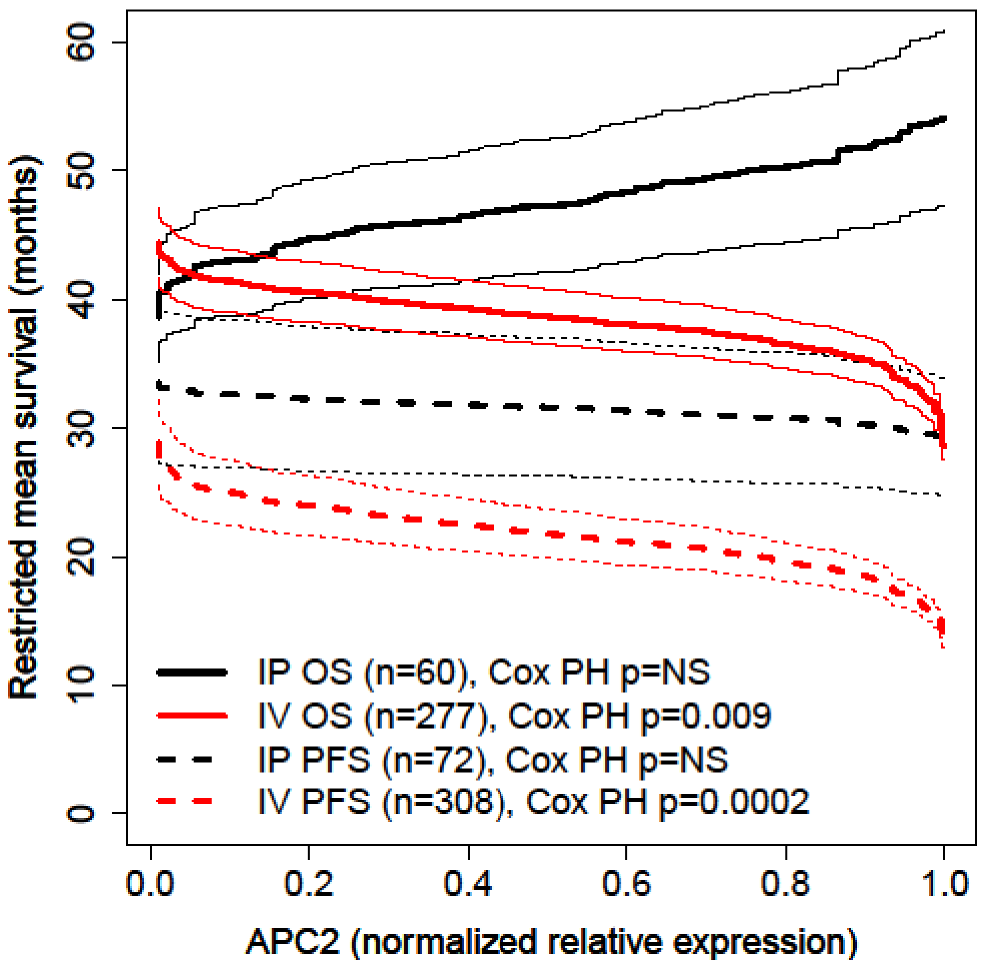

Restricted mean survival (months)
0
10
20
30
40
50
60
0.0
0.2
0.4
0.6
0.8
1.0
APC2 (normalized relative expression)
IP OS (n=60), Cox PH p=NS
IV OS (n=277), Cox PH p=0.009
IP PFS (n=72), Cox PH p=NS
IV PFS (n=308), Cox PH p=0.0002

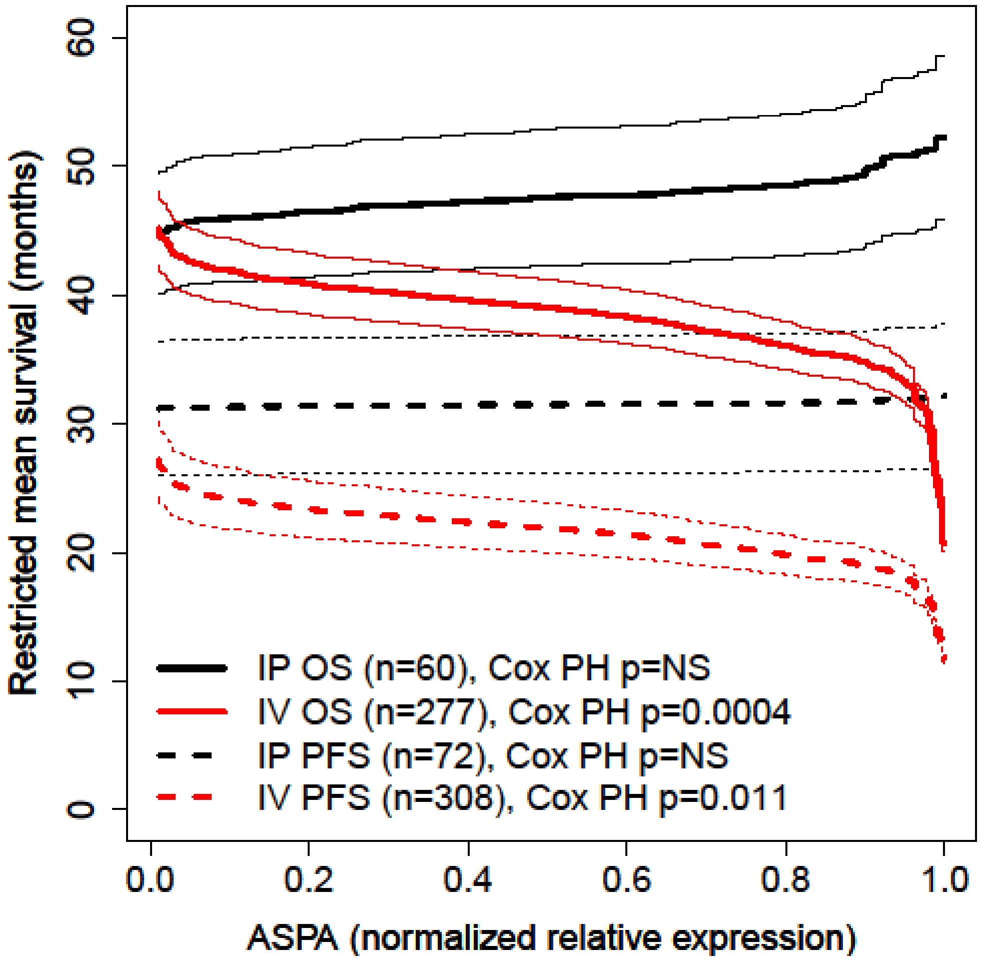

Restricted mean survival (months)
0
10
20
30
40
50
60
0.0
0.2
0.4
0.6
0.8
1.0
ASPA (normalized relative expression)
IP OS (n=60), Cox PH p=NS
IV OS (n=277), Cox PH p=0.0004
IP PFS (n=72), Cox PH p=NS
IV PFS (n=308), Cox PH p=0.011

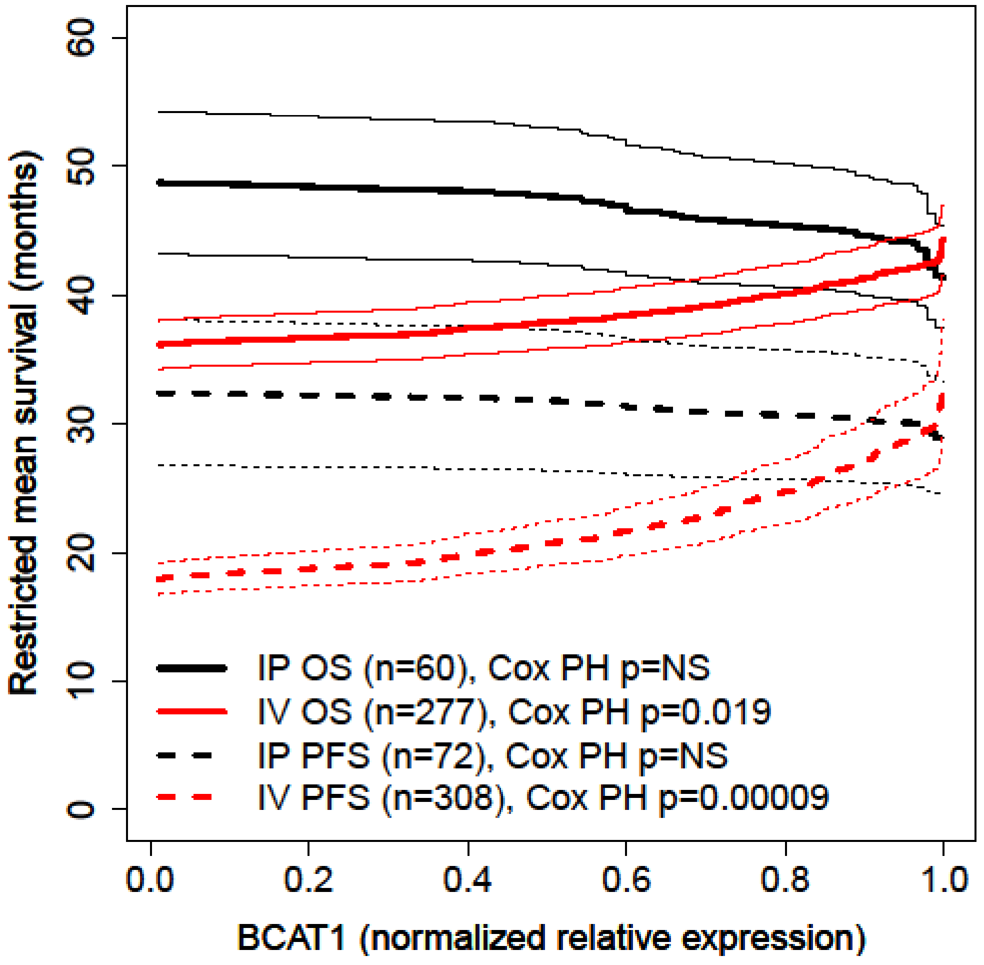

Restricted mean survival (months)
0
10
20
30
40
50
60
0.0
0.2
0.4
0.6
0.8
1.0
BCAT1 (normalized relative expression)
IP OS (n=60), Cox PH p=NS
IV OS (n=277), Cox PH p=0.019
IP PFS (n=72), Cox PH p=NS
IV PFS (n=308), Cox PH p=0.00009

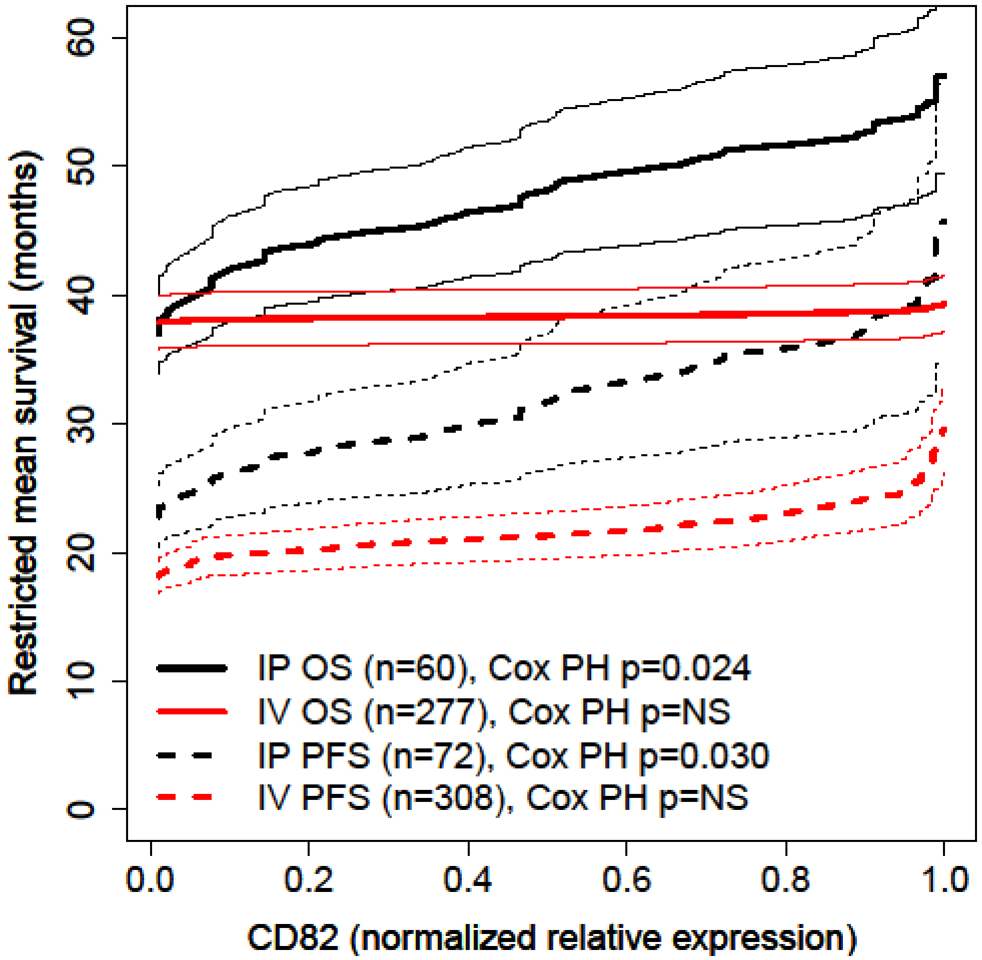

Restricted mean survival (months)
0
10
20
30
40
50
60
0.0
0.2
0.4
0.6
0.8
1.0
CD82 (normalized relative expression)
IP OS (n=60), Cox PH p=0.024
IV OS (n=277), Cox PH p=NS
IP PFS (n=72), Cox PH p=0.030
IV PFS (n=308), Cox PH p=NS

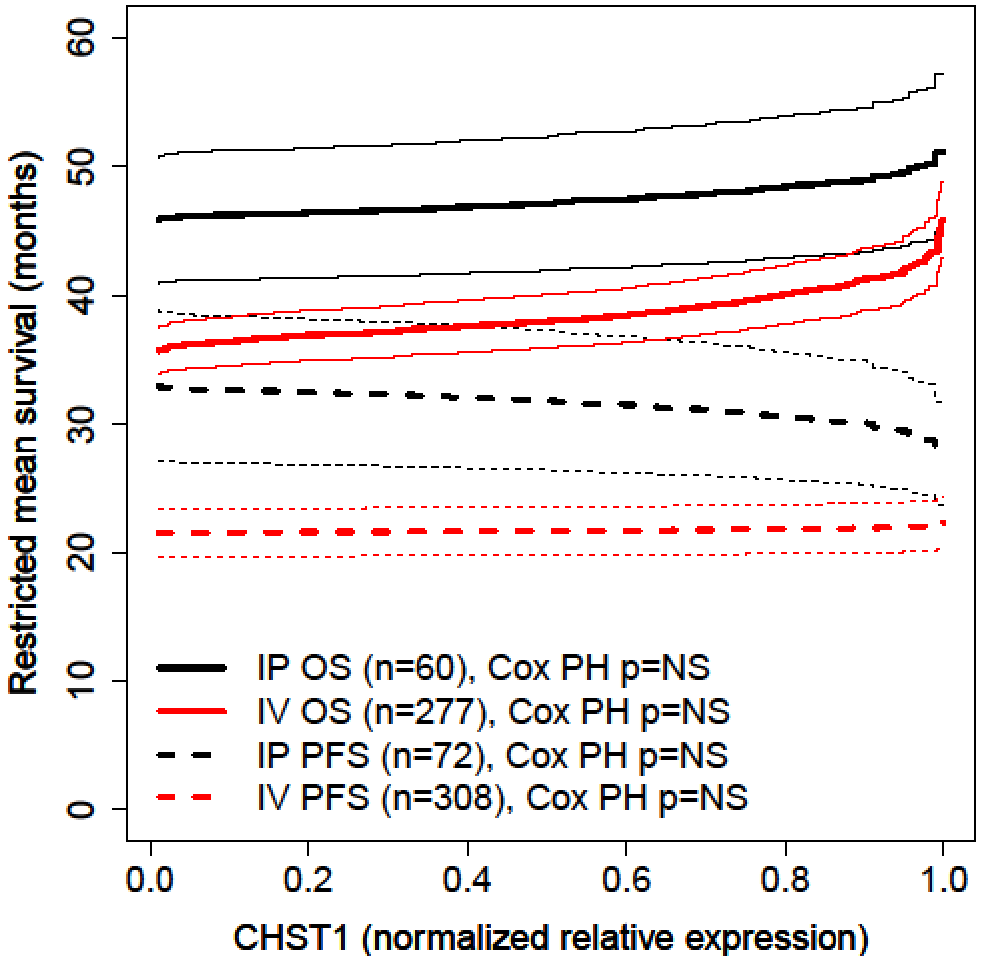

Restricted mean survival (months)
0
10
20
30
40
50
60
0.0
0.2
0.4
0.6
0.8
1.0
CHST1 (normalized relative expression)
IP OS (n=60), Cox PH p=NS
IV OS (n=277), Cox PH p=NS
IP PFS (n=72), Cox PH p=NS
IV PFS (n=308), Cox PH p=NS

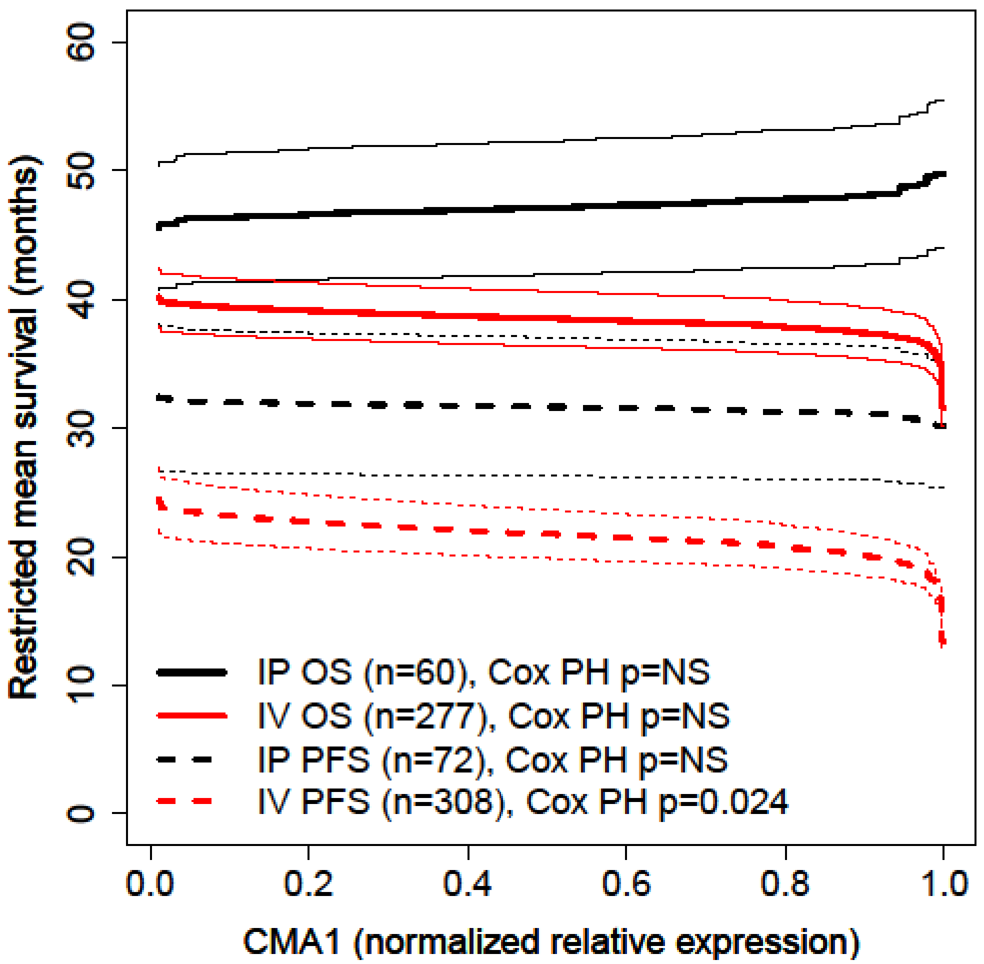
Restricted mean survival (months)
0
10
20
30
40
50
60
0.0
0.2
0.4
0.6
0.8
1.0
CMA1 (normalized relative expression)
IP OS (n=60), Cox PH p=NS
IV OS (n=277), Cox PH p=NS
IP PFS (n=72), Cox PH p=NS
IV PFS (n=308), Cox PH p=0.024

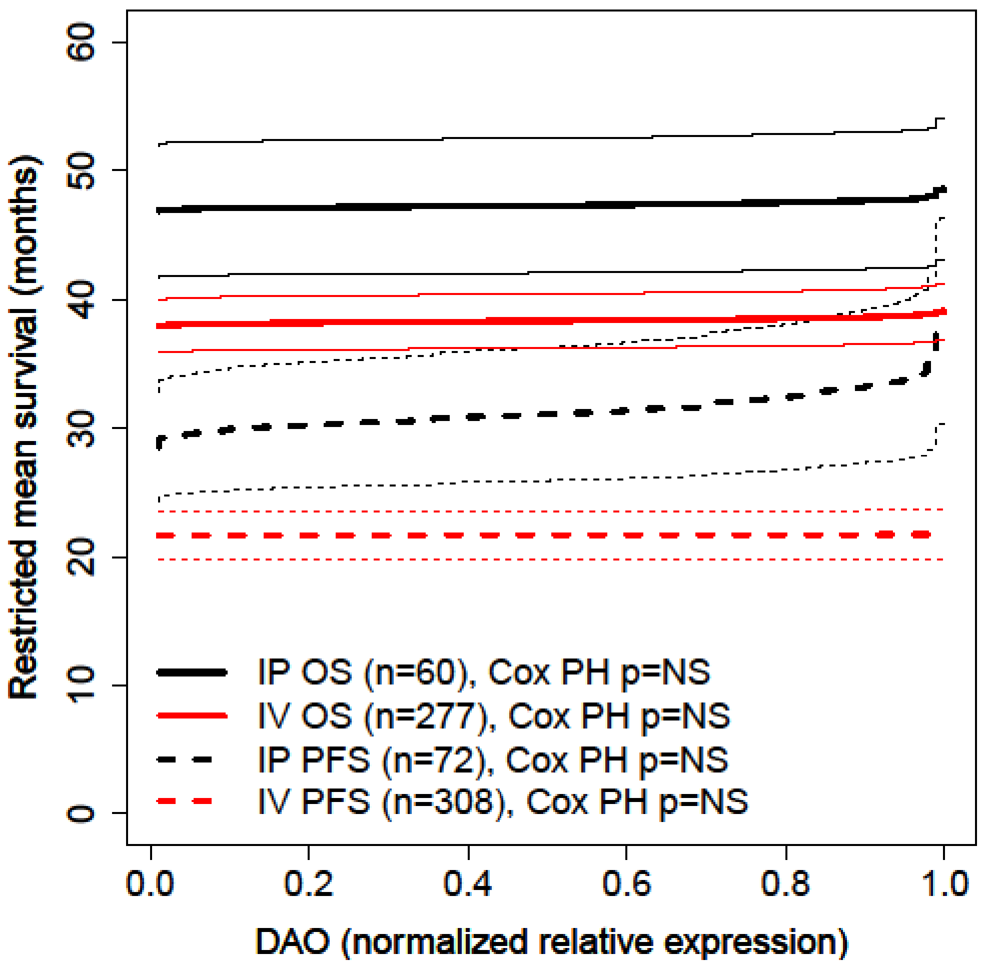

Restricted mean survival (months)
0
10
20
30
40
50
60
0.0
0.2
0.4
0.6
0.8
1.0
DAO (normalized relative expression)
IP OS (n=60), Cox PH p=NS
IV OS (n=277), Cox PH p=NS
IP PFS (n=72), Cox PH p=NS
IV PFS (n=308), Cox PH p=NS

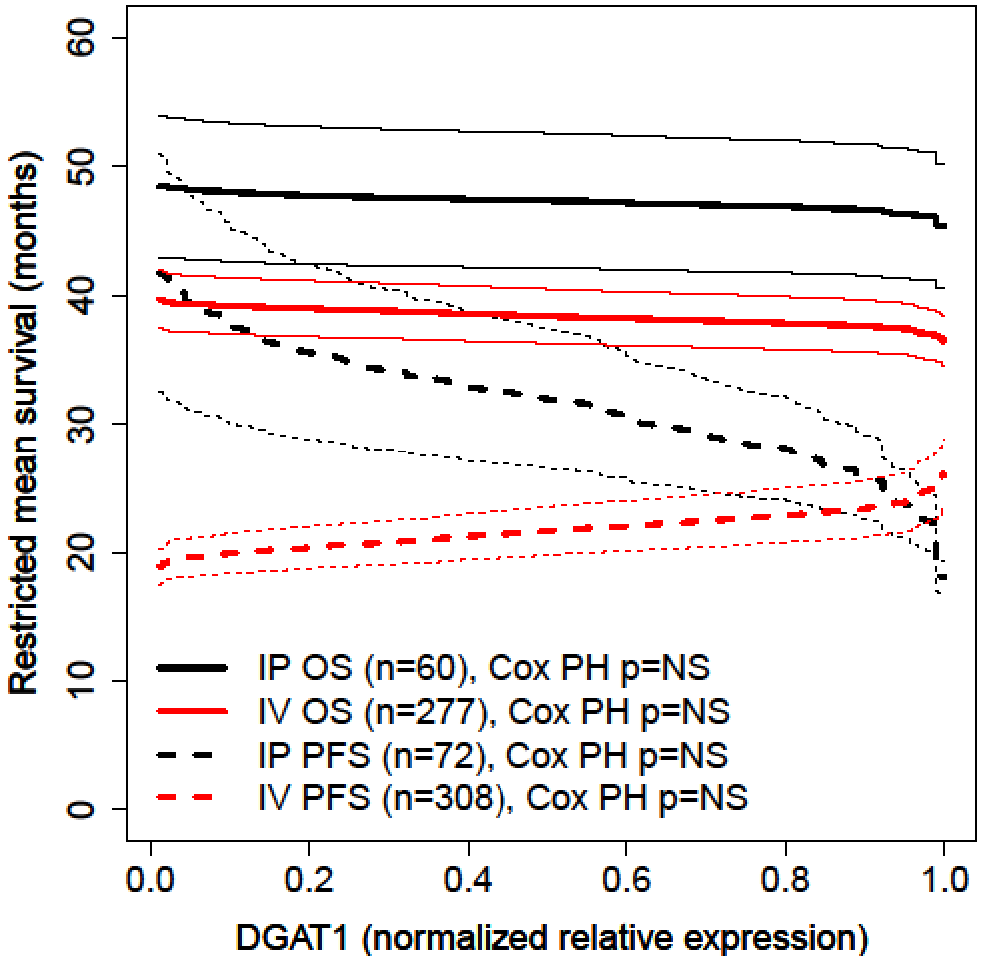

Restricted mean survival (months)
0
10
20
30
40
50
60
0.0
0.2
0.4
0.6
0.8
1.0
DGAT1 (normalized relative expression)
IP OS (n=60), Cox PH p=NS
IV OS (n=277), Cox PH p=NS
IP PFS (n=72), Cox PH p=NS
IV PFS (n=308), Cox PH p=NS

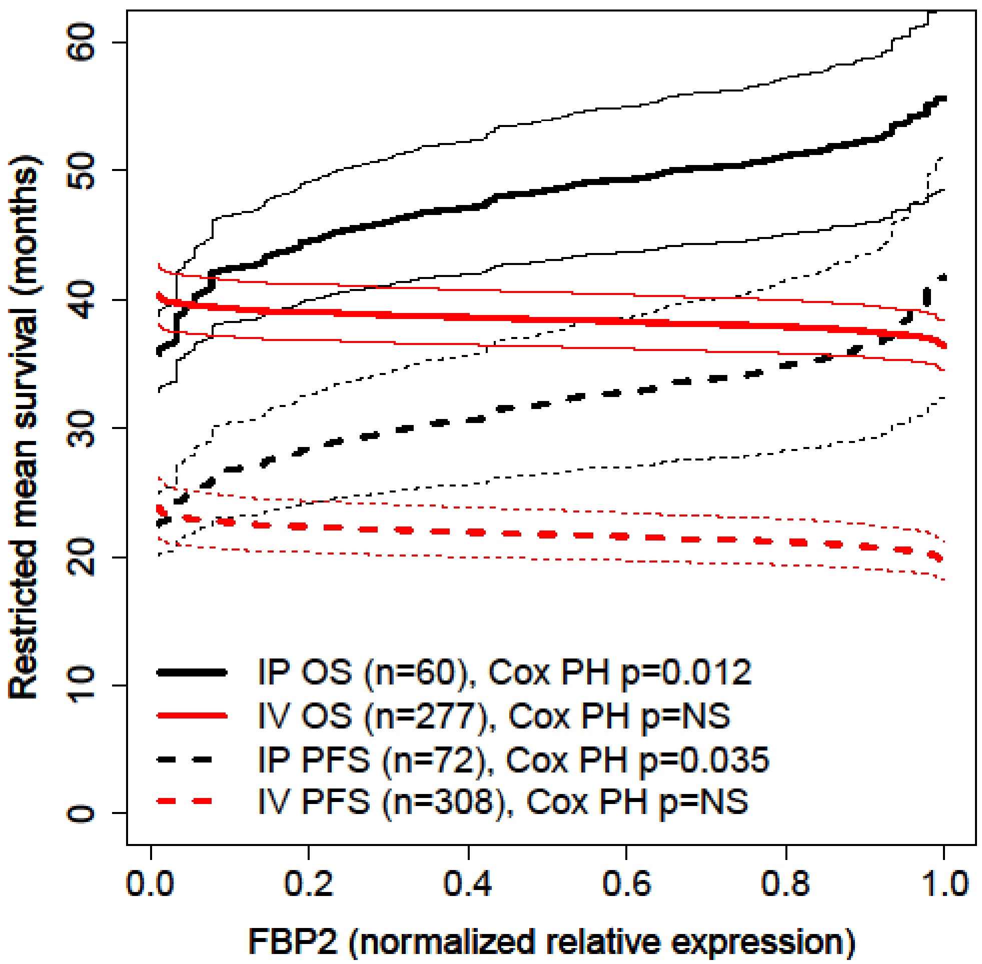

Restricted mean survival (months)
0
10
20
30
40
50
60
0.0
0.2
0.4
0.6
0.8
1.0
FBP2 (normalized relative expression)
IP OS (n=60), Cox PH p=0.012
IV OS (n=277), Cox PH p=NS
IP PFS (n=72), Cox PH p=0.035
IV PFS (n=308), Cox PH p=NS

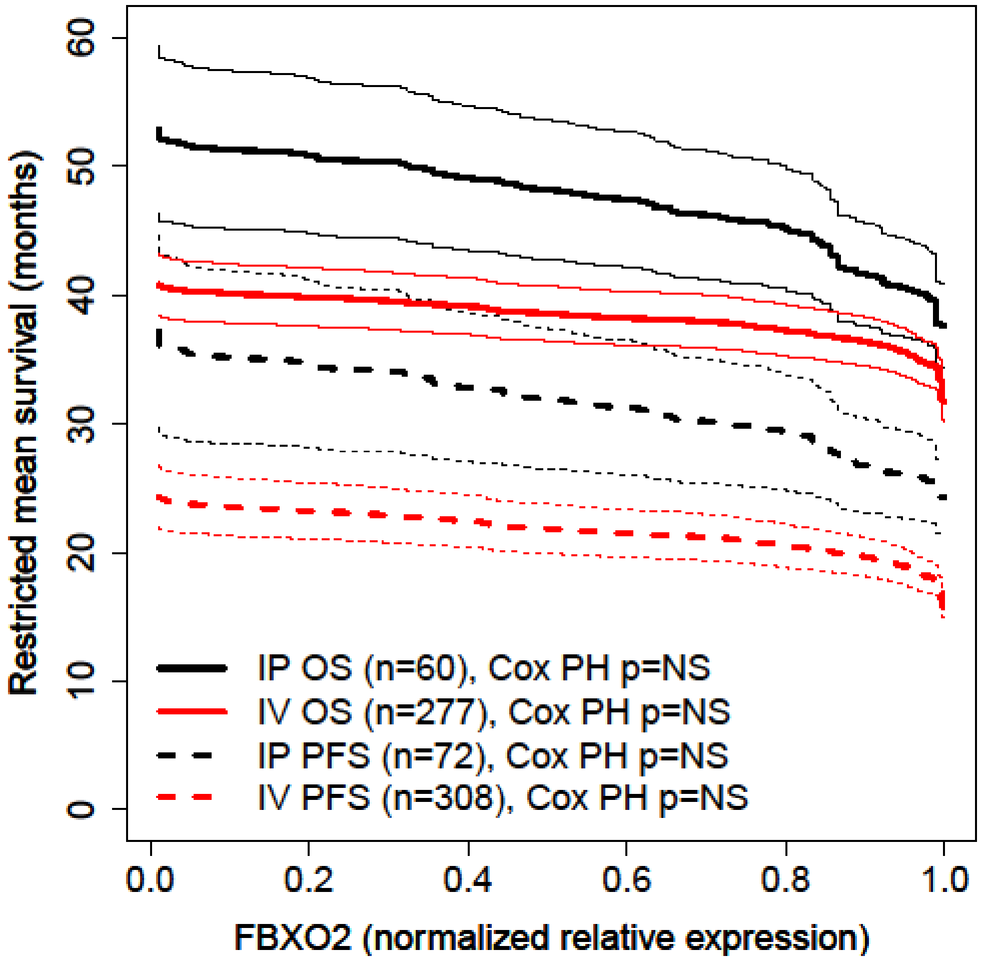

Restricted mean survival (months)
0
10
20
30
40
50
60
0.0
0.2
0.4
0.6
0.8
1.0
FBXO2 (normalized relative expression)
IP OS (n=60), Cox PH p=NS
IV OS (n=277), Cox PH p=NS
IP PFS (n=72), Cox PH p=NS
IV PFS (n=308), Cox PH p=NS

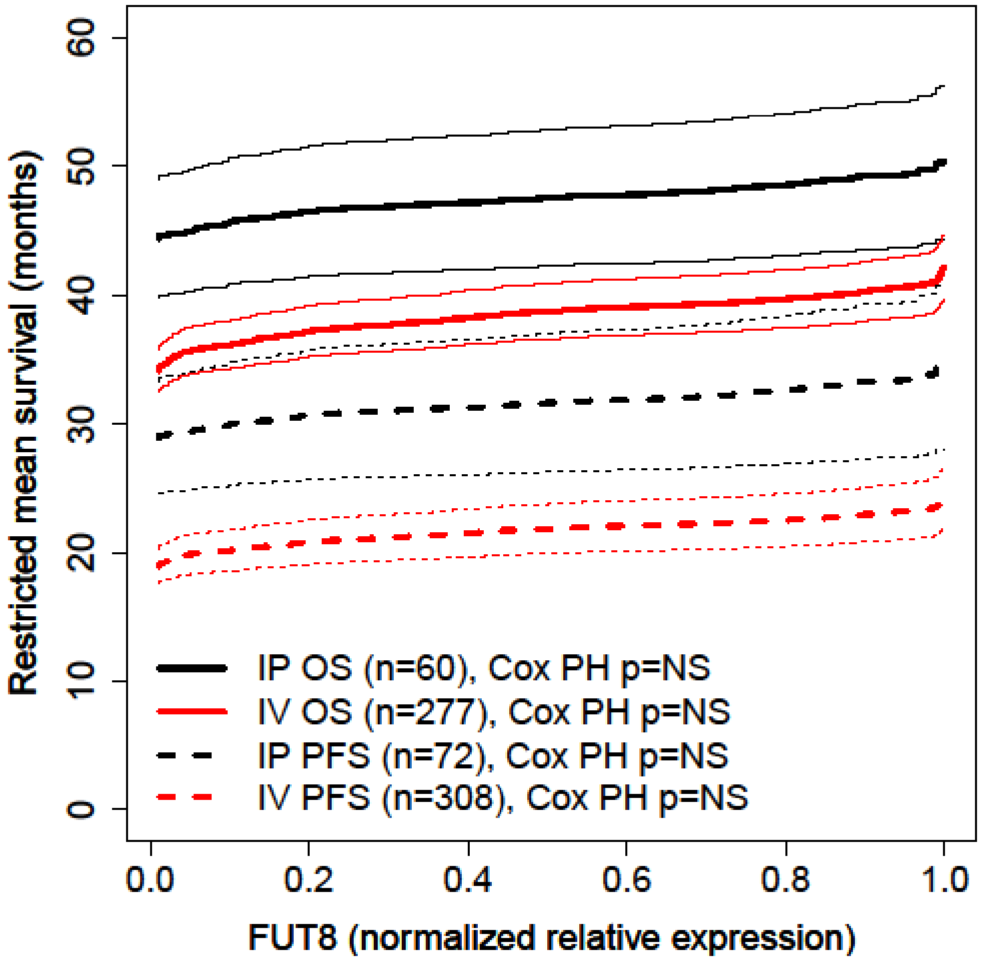

Restricted mean survival (months)
0
10
20
30
40
50
60
0.0
0.2
0.4
0.6
0.8
1.0
FUT8 (normalized relative expression)
IP OS (n=60), Cox PH p=NS
IV OS (n=277), Cox PH p=NS
IP PFS (n=72), Cox PH p=NS
IV PFS (n=308), Cox PH p=NS

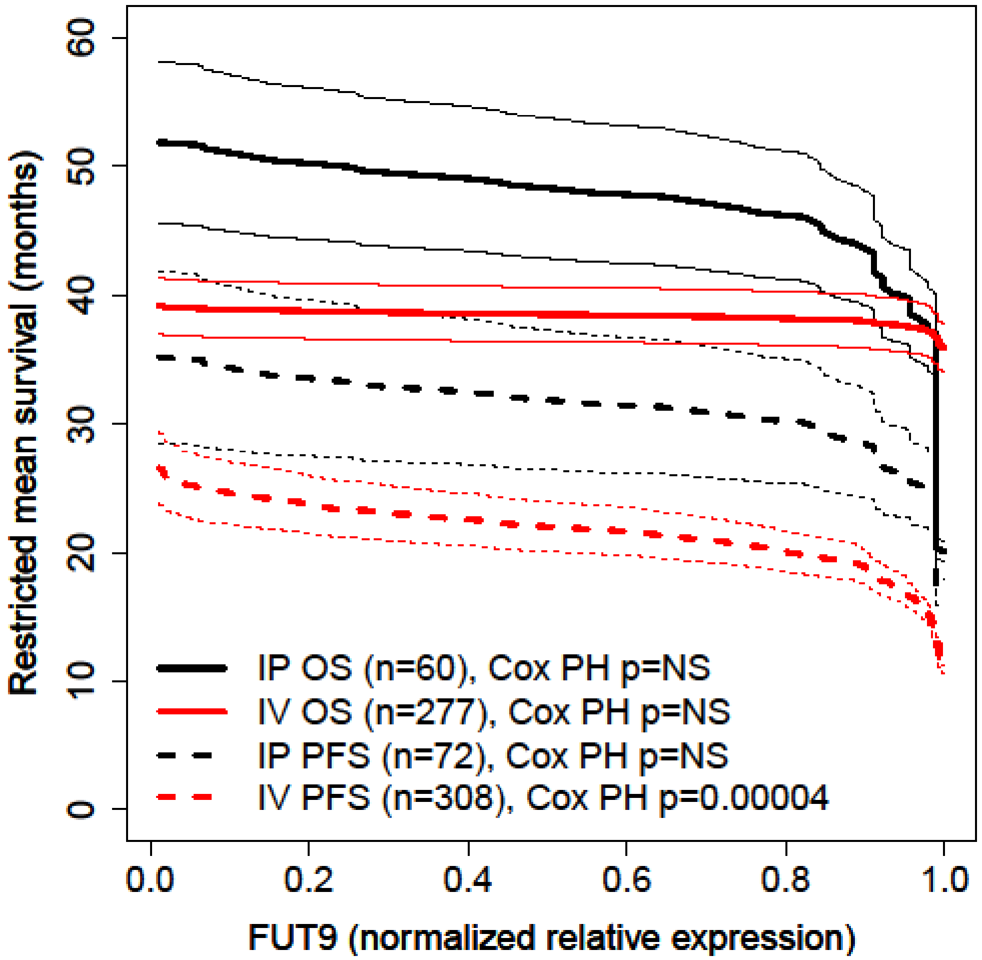
Restricted mean survival (months)
0
10
20
30
40
50
60
0.0
0.2
0.4
0.6
0.8
1.0
FUT9 (normalized relative expression)
IP OS (n=60), Cox PH p=NS
IV OS (n=277), Cox PH p=NS
IP PFS (n=72), Cox PH p=NS
IV PFS (n=308), Cox PH p=0.00004

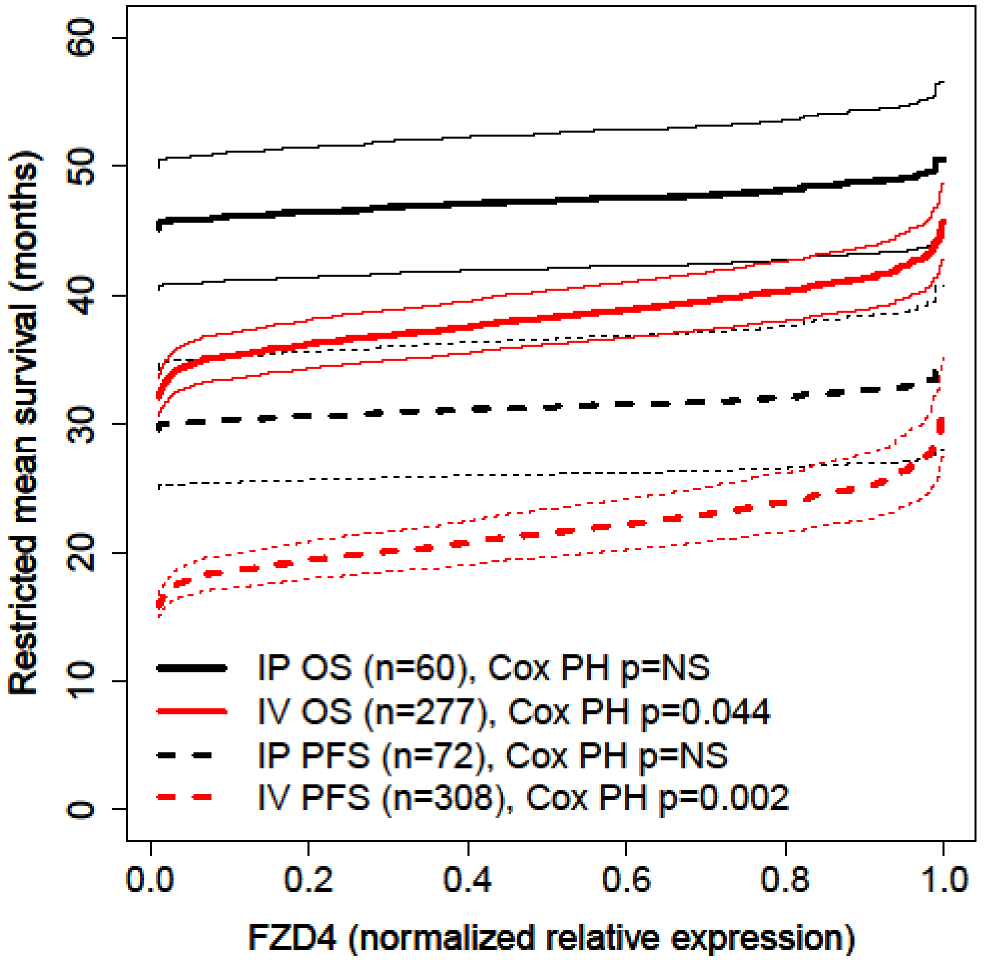
Restricted mean survival (months)
0
10
20
30
40
50
60
0.0
0.2
0.4
0.6
0.8
1.0
FZD4 (normalized relative expression)
IP OS (n=60), Cox PH p=NS
IV OS (n=277), Cox PH p=0.044
IP PFS (n=72), Cox PH p=NS
IV PFS (n=308), Cox PH p=0.002

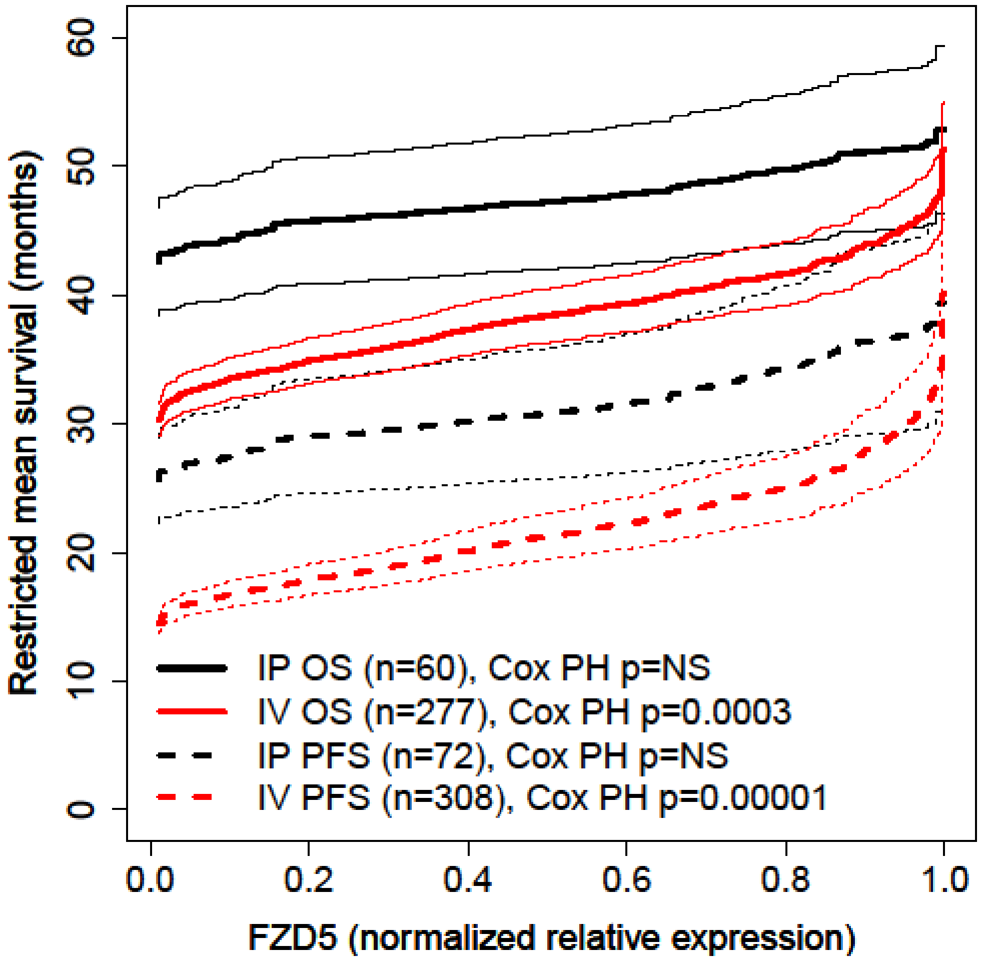
60
50
40
30
20
10
0
Restricted mean survival (months)
0.0
0.2
0.4
0.6
0.8
1.0
FZD5 (normalized relative expression)
IP OS (n=60), Cox PH p=NS
IV OS (n=277), Cox PH p=0.0003
IP PFS (n=72), Cox PH p=NS
IV PFS (n=308), Cox PH p=0.00001

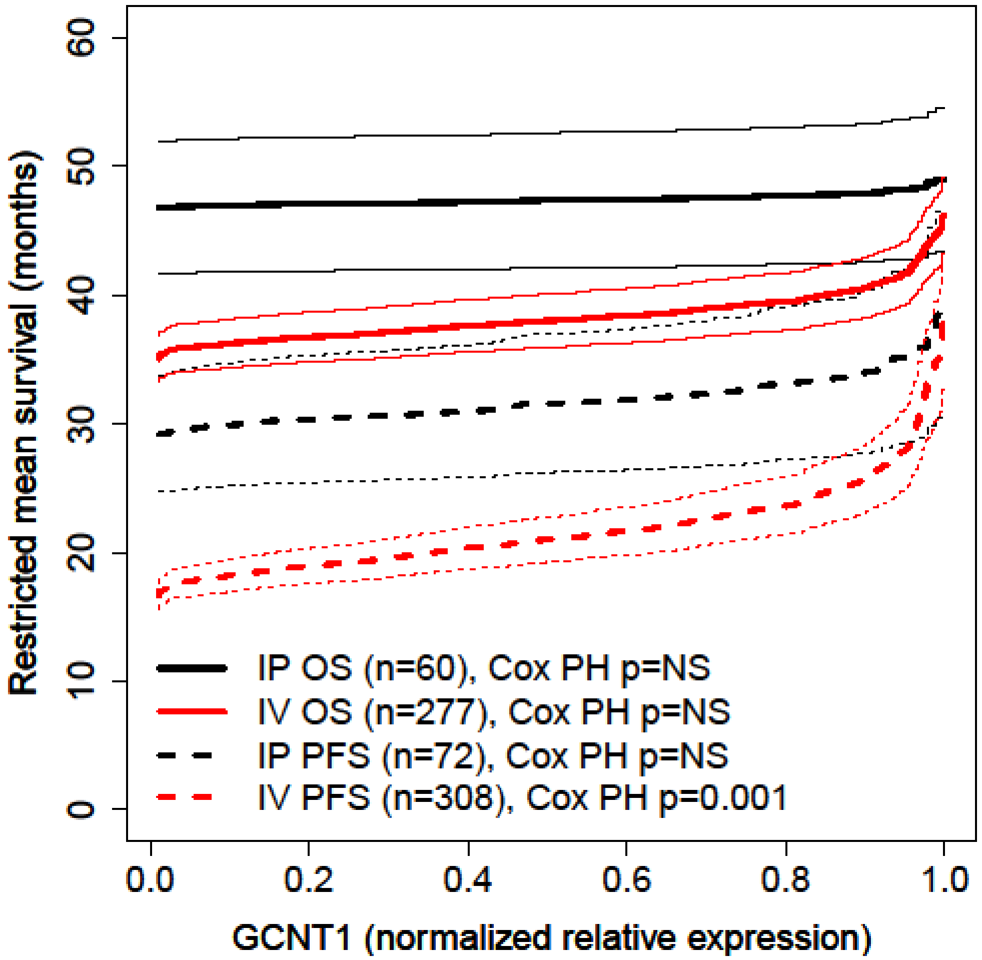
Restricted mean survival (months)
0
10
20
30
40
50
60
0.0
0.2
0.4
0.6
0.8
1.0
GCNT1 (normalized relative expression)
IP OS (n=60), Cox PH p=NS
IV OS (n=277), Cox PH p=NS
IP PFS (n=72), Cox PH p=NS
IV PFS (n=308), Cox PH p=0.001

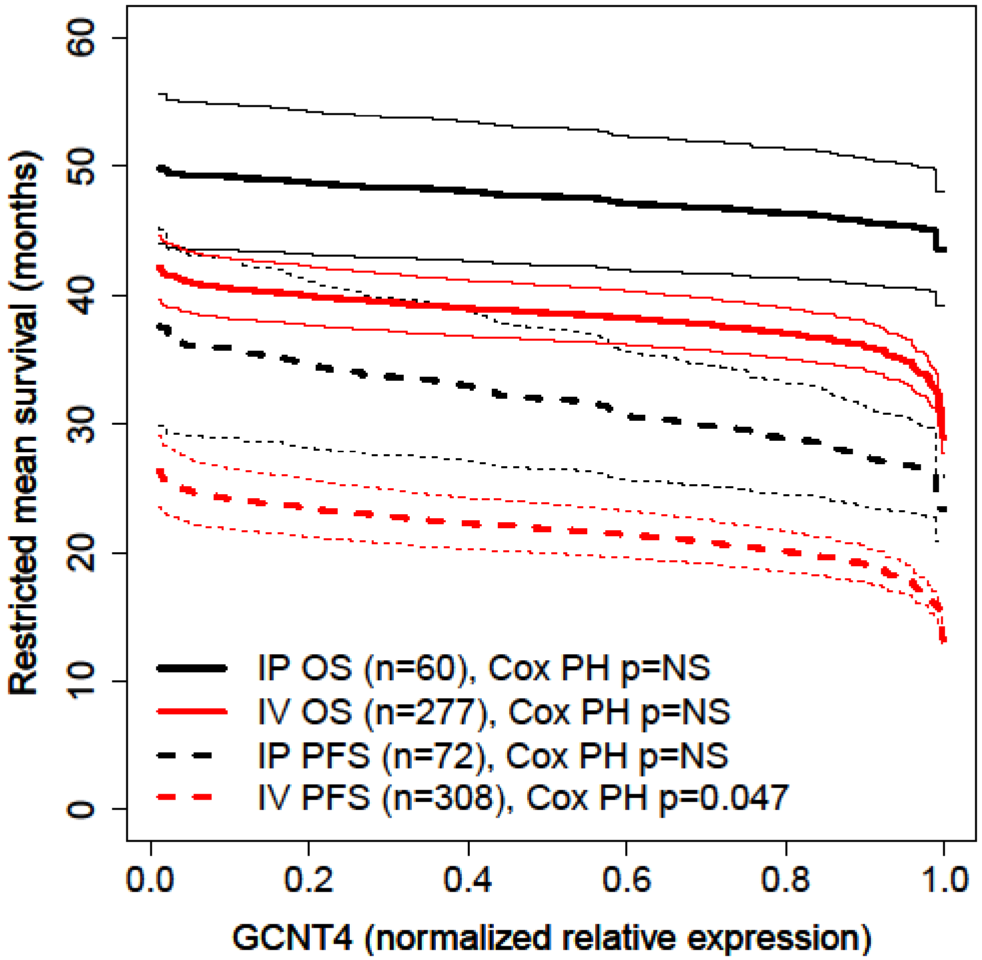

Restricted mean survival (months)
0
10
20
30
40
50
60
0.0
0.2
0.4
0.6
0.8
1.0
GCNT4 (normalized relative expression)
IP OS (n=60), Cox PH p=NS
IV OS (n=277), Cox PH p=NS
IP PFS (n=72), Cox PH p=NS
IV PFS (n=308), Cox PH p=0.047

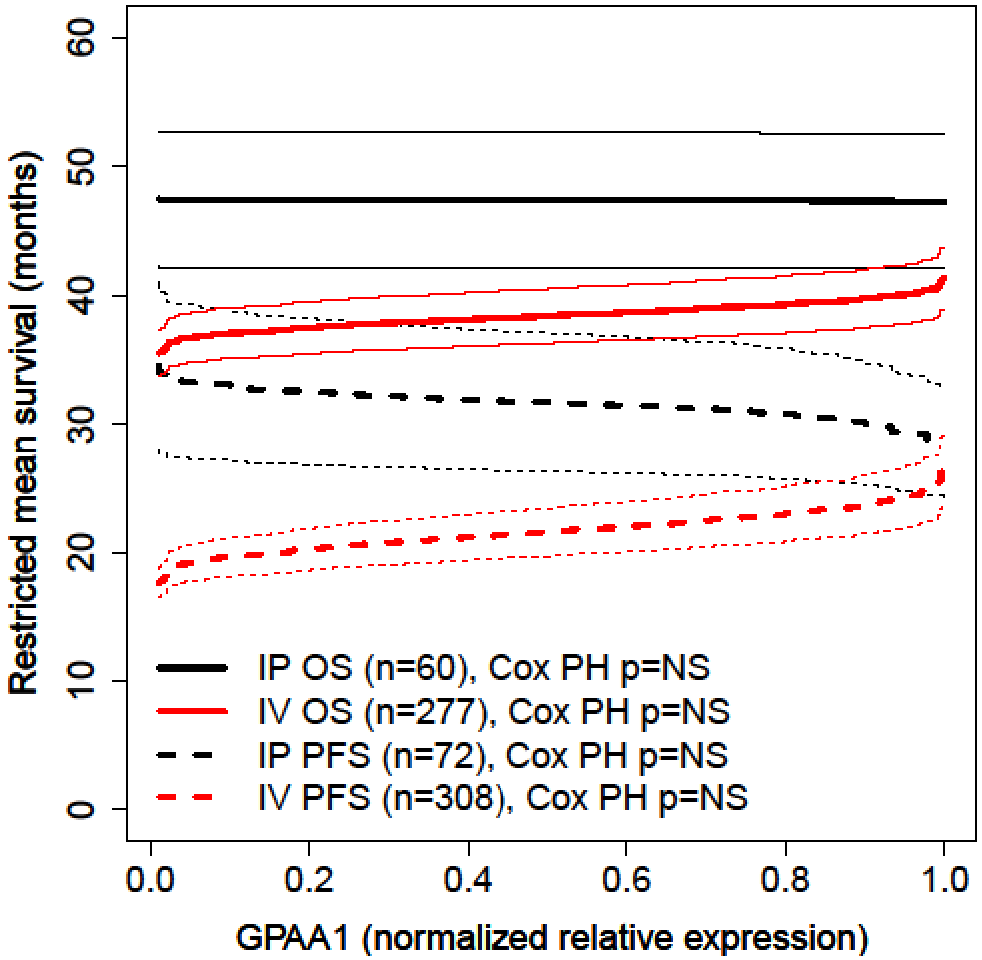

Restricted mean survival (months)
0
10
20
30
40
50
60
0.0
0.2
0.4
0.6
0.8
1.0
GPAA1 (normalized relative expression)
IP OS (n=60), Cox PH p=NS
IV OS (n=277), Cox PH p=NS
IP PFS (n=72), Cox PH p=NS
IV PFS (n=308), Cox PH p=NS

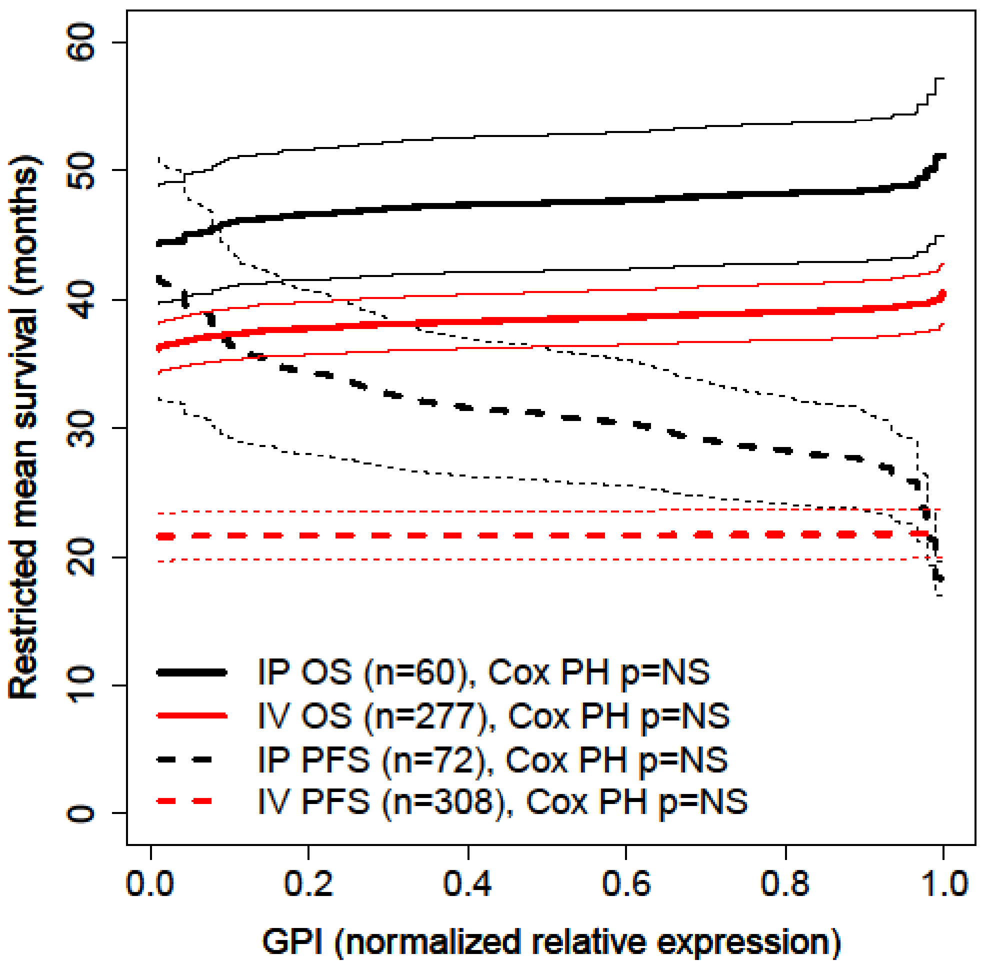
Restricted mean survival (months)
0
10
20
30
40
50
60
0.0
0.2
0.4
0.6
0.8
1.0
GPI (normalized relative expression)
IP OS (n=60), Cox PH p=NS
IV OS (n=277), Cox PH p=NS
IP PFS (n=72), Cox PH p=NS
IV PFS (n=308), Cox PH p=NS

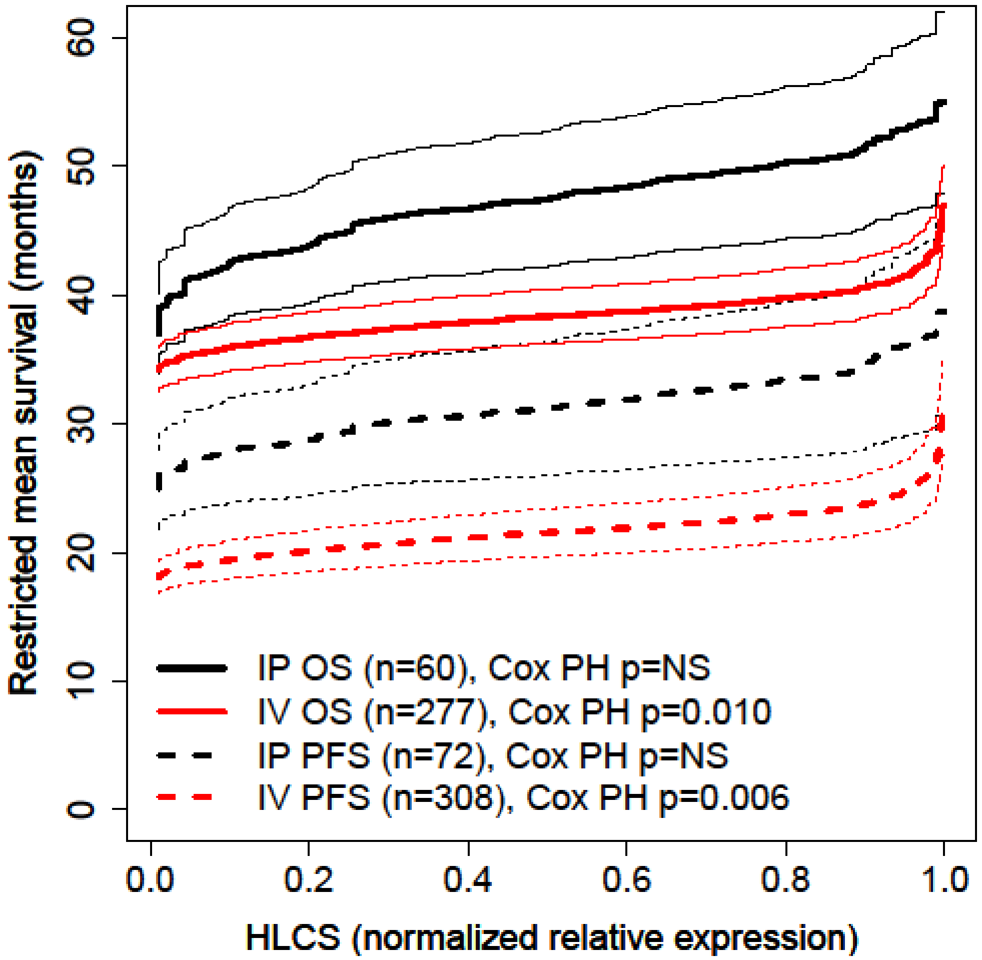

Restricted mean survival (months)
0
10
20
30
40
50
60
0.0
0.2
0.4
0.6
0.8
1.0
HLCS (normalized relative expression)
IP OS (n=60), Cox PH p=NS
IV OS (n=277), Cox PH p=0.010
IP PFS (n=72), Cox PH p=NS
IV PFS (n=308), Cox PH p=0.006

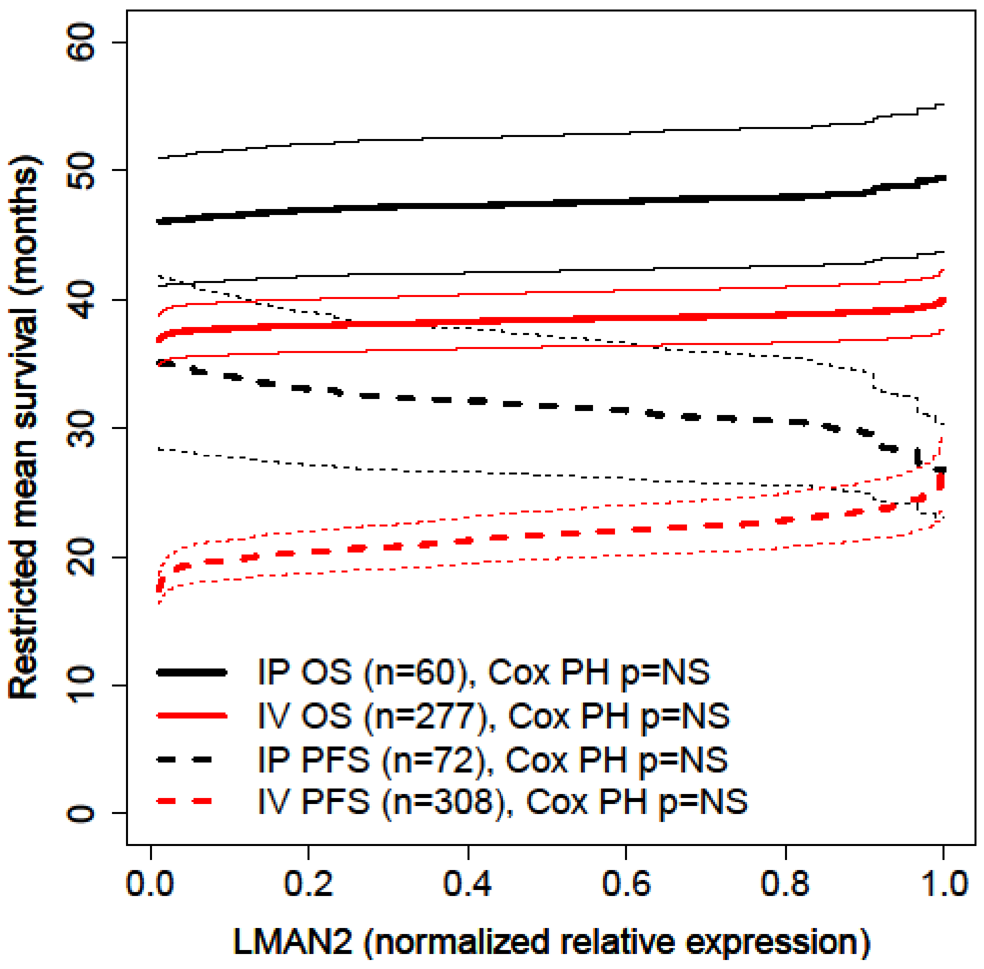

Restricted mean survival (months)
0
10
20
30
40
50
60
0.0
0.2
0.4
0.6
0.8
1.0
LMAN2 (normalized relative expression)
IP OS (n=60), Cox PH p=NS
IV OS (n=277), Cox PH p=NS
IP PFS (n=72), Cox PH p=NS
IV PFS (n=308), Cox PH p=NS

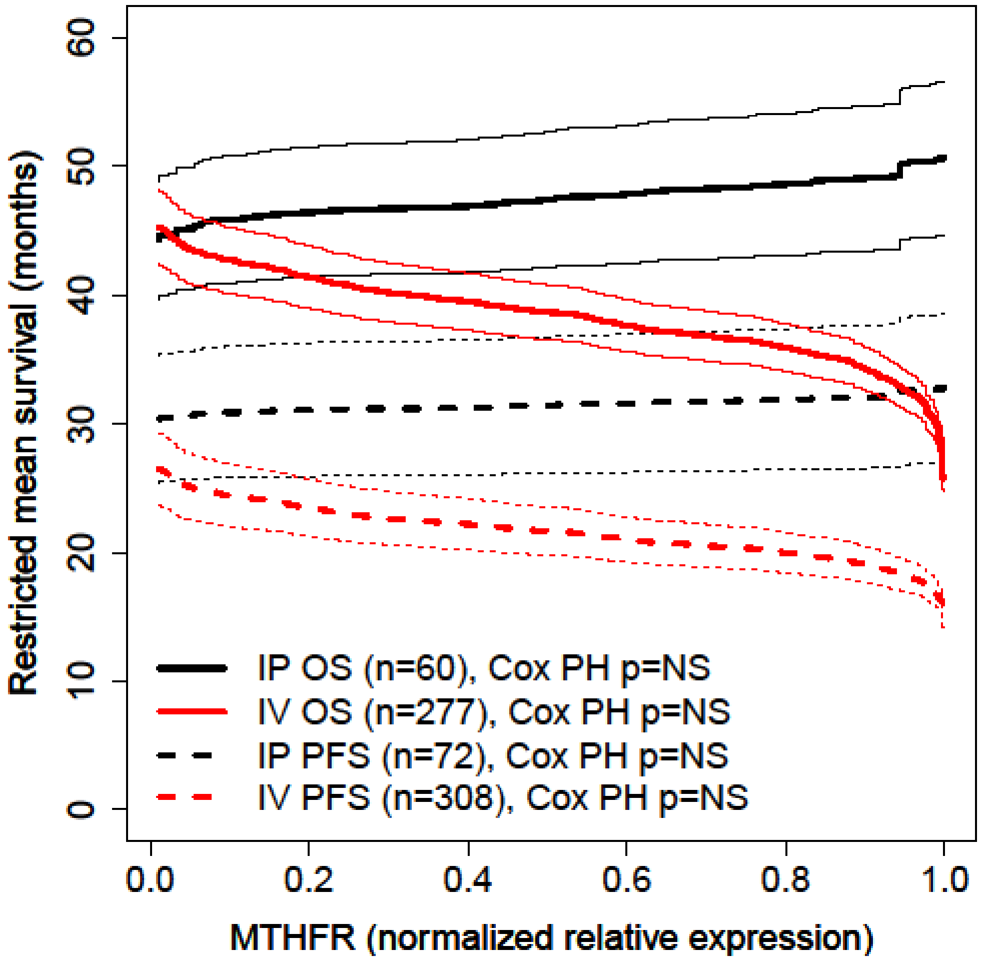

Restricted mean survival (months)
0
10
20
30
40
50
60
0.0
0.2
0.4
0.6
0.8
1.0
MTHFR (normalized relative expression)
IP OS (n=60), Cox PH p=NS
IV OS (n=277), Cox PH p=NS
IP PFS (n=72), Cox PH p=NS
IV PFS (n=308), Cox PH p=NS

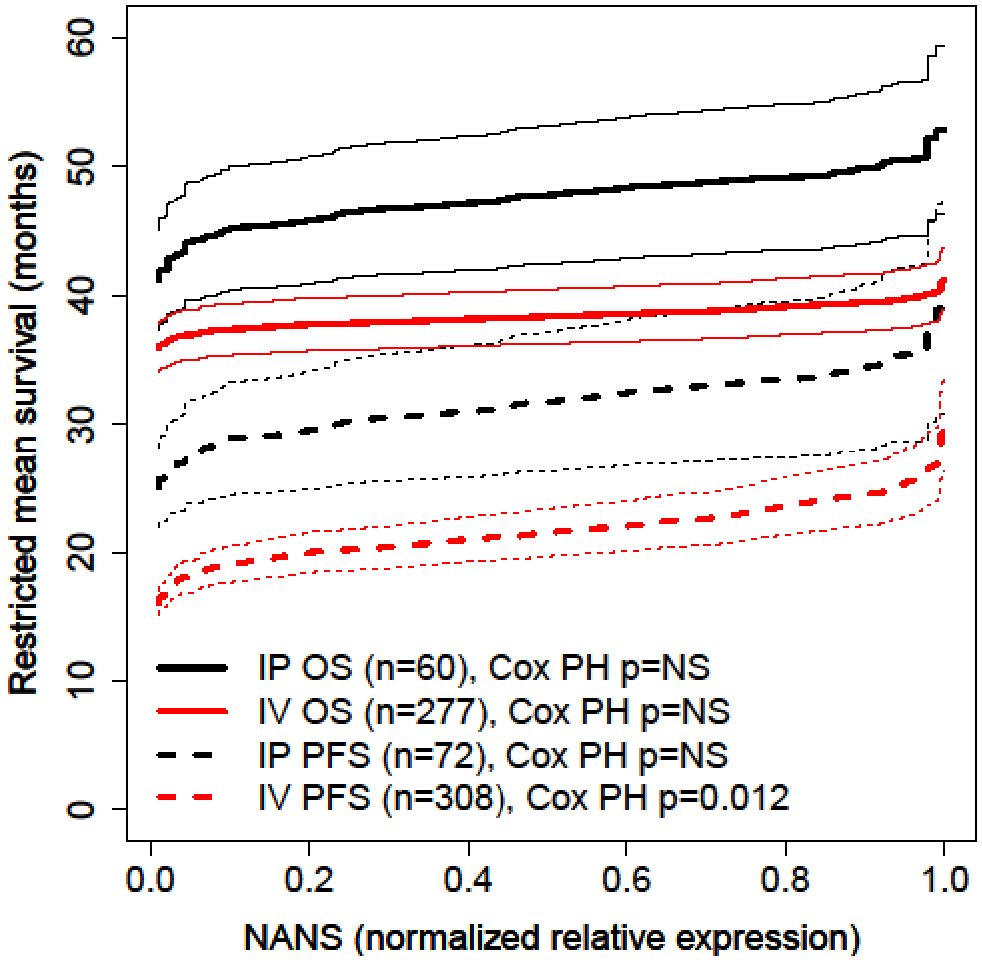
Restricted mean survival (months)
0
10
20
30
40
50
60
0.0
0.2
0.4
0.6
0.8
1.0
NANS (normalized relative expression)
IP OS (n=60), Cox PH p=NS
IV OS (n=277), Cox PH p=NS
IP PFS (n=72), Cox PH p=NS
IV PFS (n=308), Cox PH p=0.012

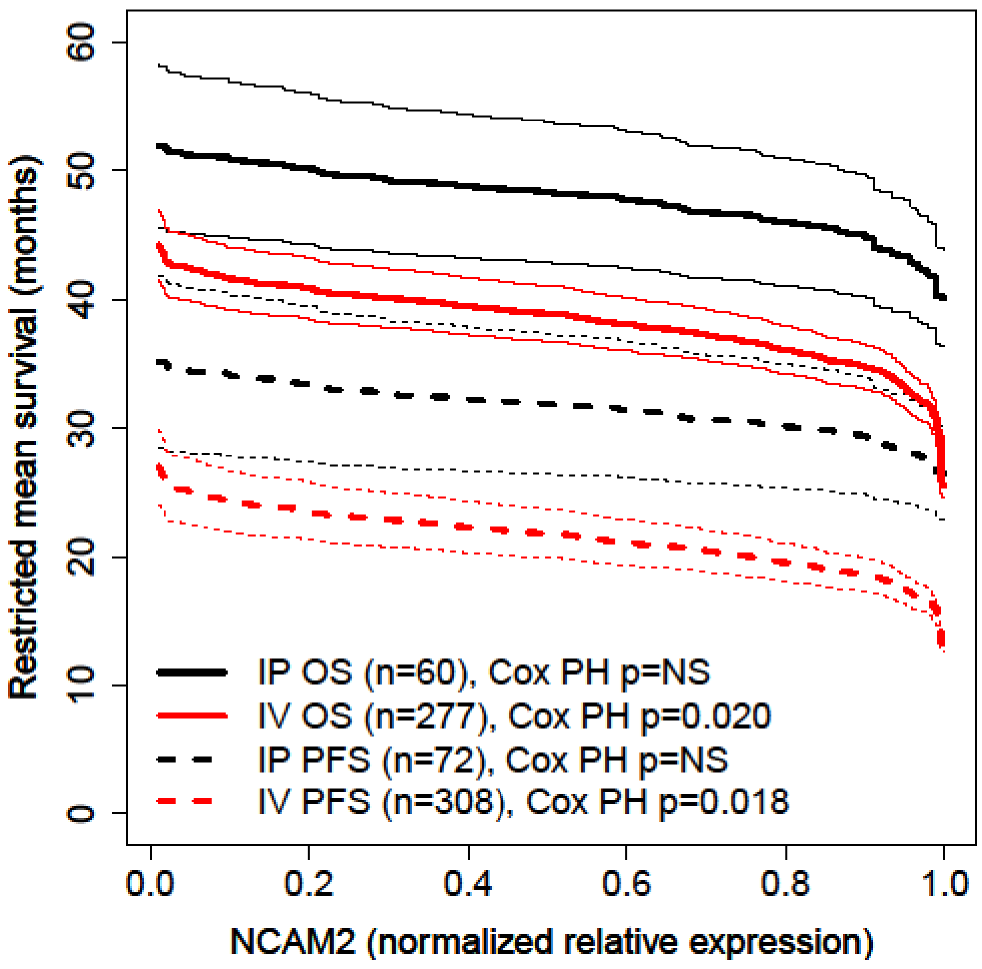
Restricted mean survival (months)
0
10
20
30
40
50
60
0.0
0.2
0.4
0.6
0.8
1.0
NCAM2 (normalized relative expression)
IP OS (n=60), Cox PH p=NS
IV OS (n=277), Cox PH p=0.020
IP PFS (n=72), Cox PH p=NS
IV PFS (n=308), Cox PH p=0.018

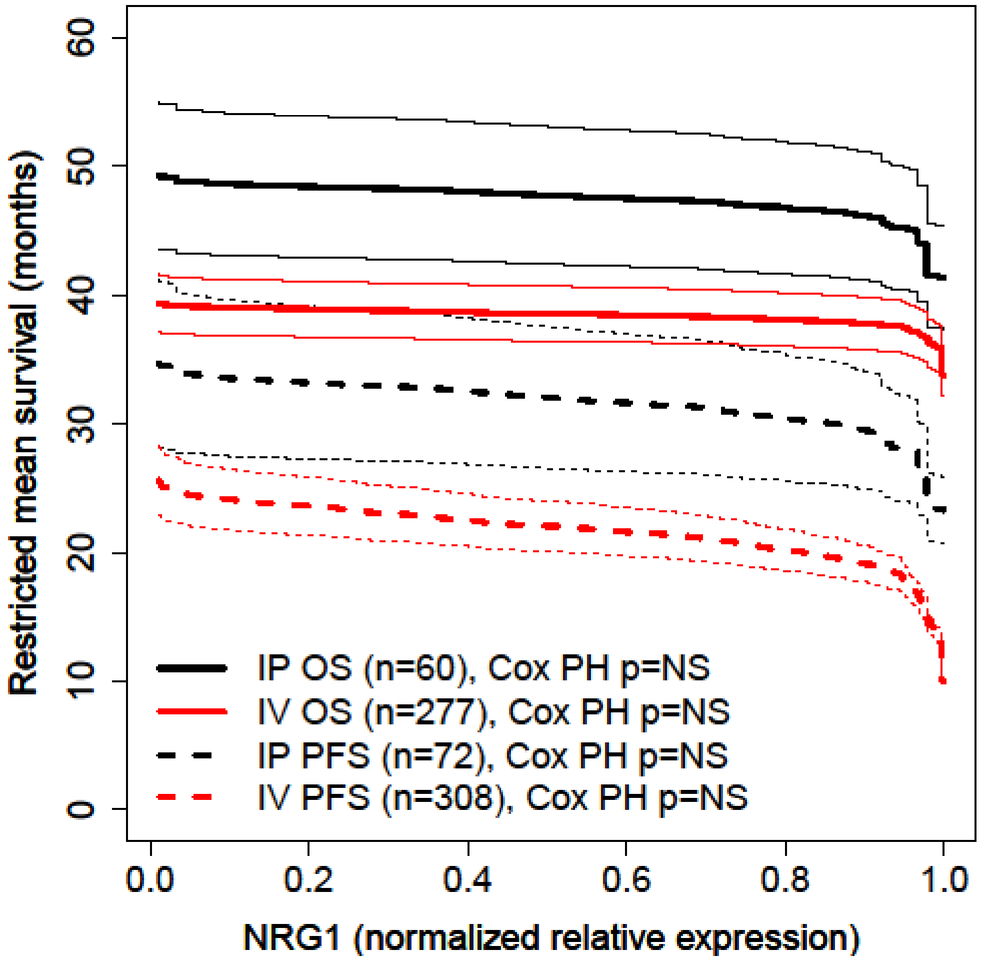

Restricted mean survival (months)
0
10
20
30
40
50
60
0.0
0.2
0.4
0.6
0.8
1.0
NRG1 (normalized relative expression)
IP OS (n=60), Cox PH p=NS
IV OS (n=277), Cox PH p=NS
IP PFS (n=72), Cox PH p=NS
IV PFS (n=308), Cox PH p=NS

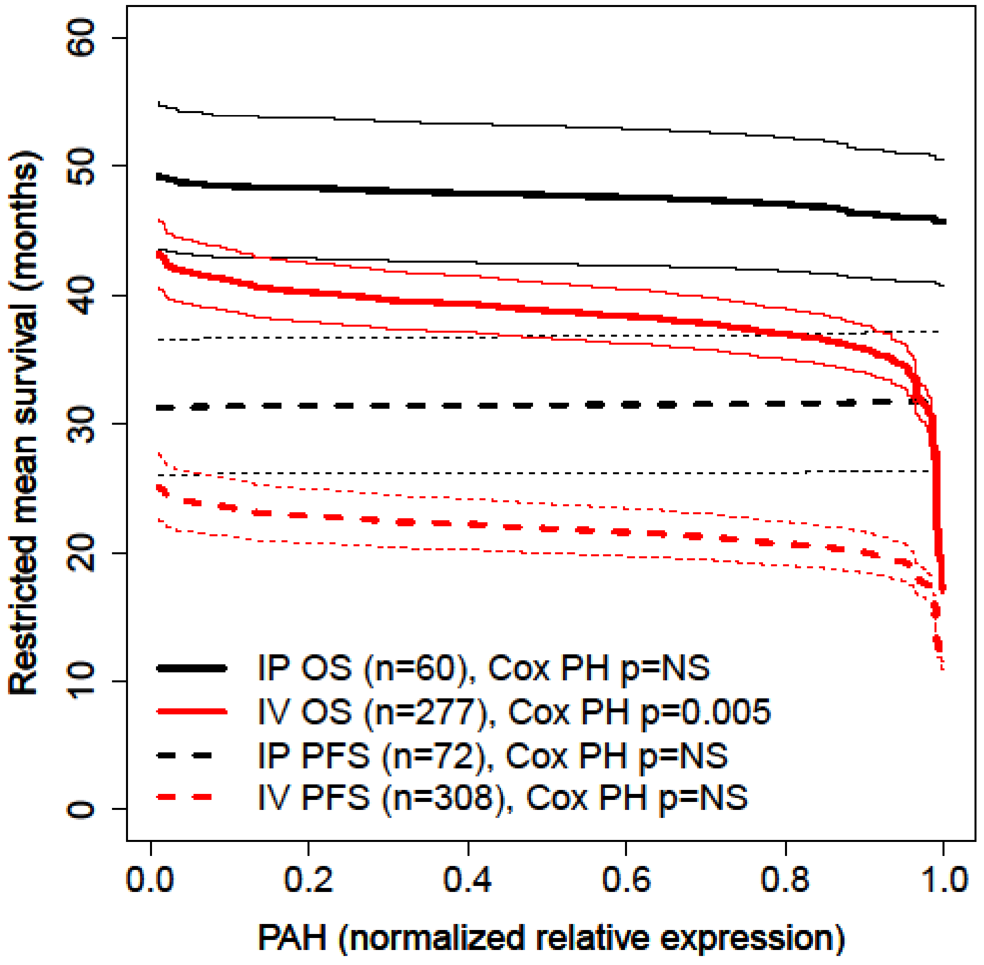

Restricted mean survival (months)
0
10
20
30
40
50
60
0.0
0.2
0.4
0.6
0.8
1.0
PAH (normalized relative expression)
IP OS (n=60), Cox PH p=NS
IV OS (n=277), Cox PH p=0.005
IP PFS (n=72), Cox PH p=NS
IV PFS (n=308), Cox PH p=NS

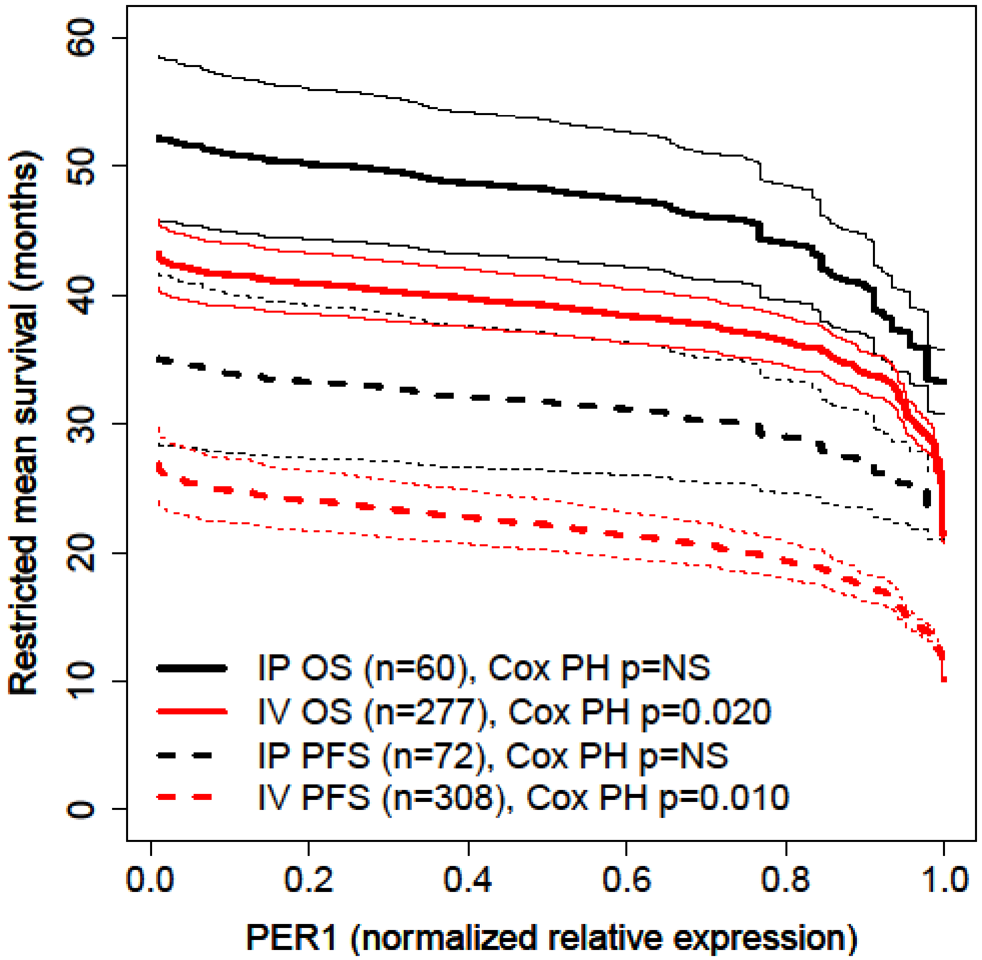

Restricted mean survival (months)
0
10
20
30
40
50
60
0.0
0.2
0.4
0.6
0.8
1.0
PER1 (normalized relative expression)
IP OS (n=60), Cox PH p=NS
IV OS (n=277), Cox PH p=0.020
IP PFS (n=72), Cox PH p=NS
IV PFS (n=308), Cox PH p=0.010

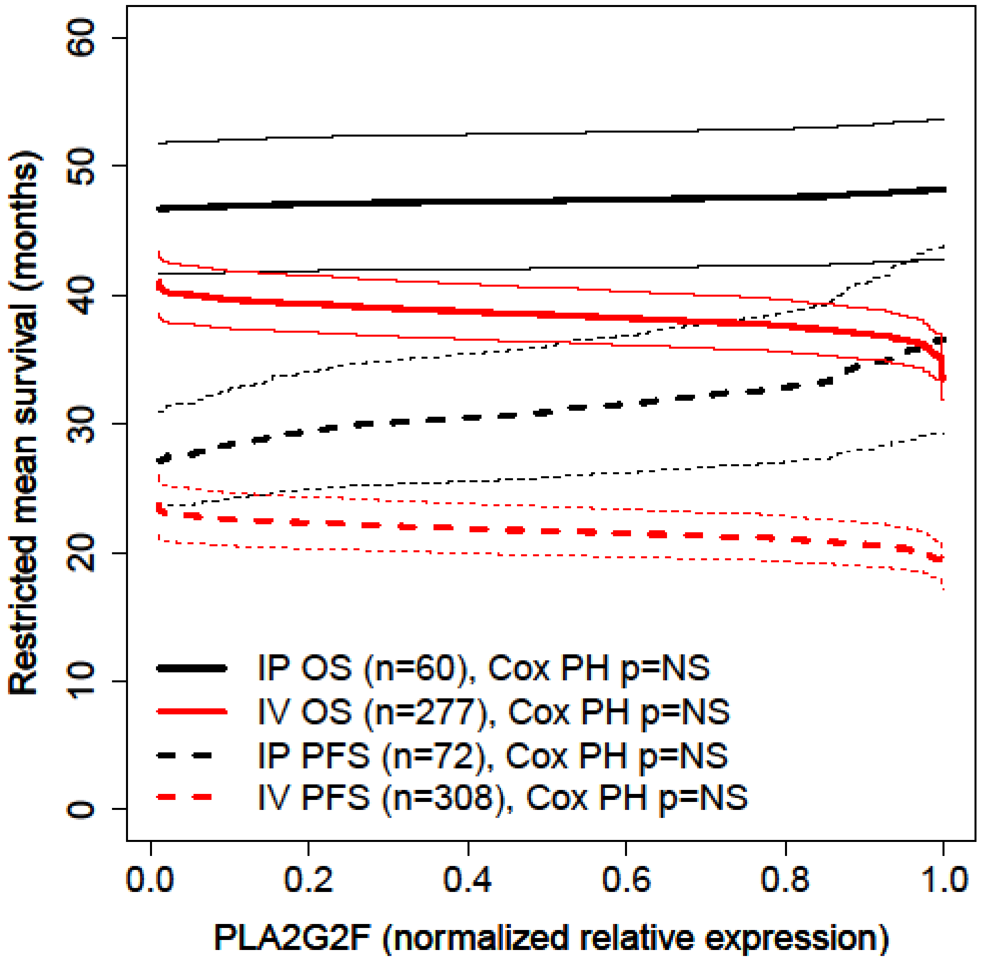

Restricted mean survival (months)
0
10
20
30
40
50
60
0.0
0.2
0.4
0.6
0.8
1.0
PLA2G2F (normalized relative expression)
IP OS (n=60), Cox PH p=NS
IV OS (n=277), Cox PH p=NS
IP PFS (n=72), Cox PH p=NS
IV PFS (n=308), Cox PH p=NS

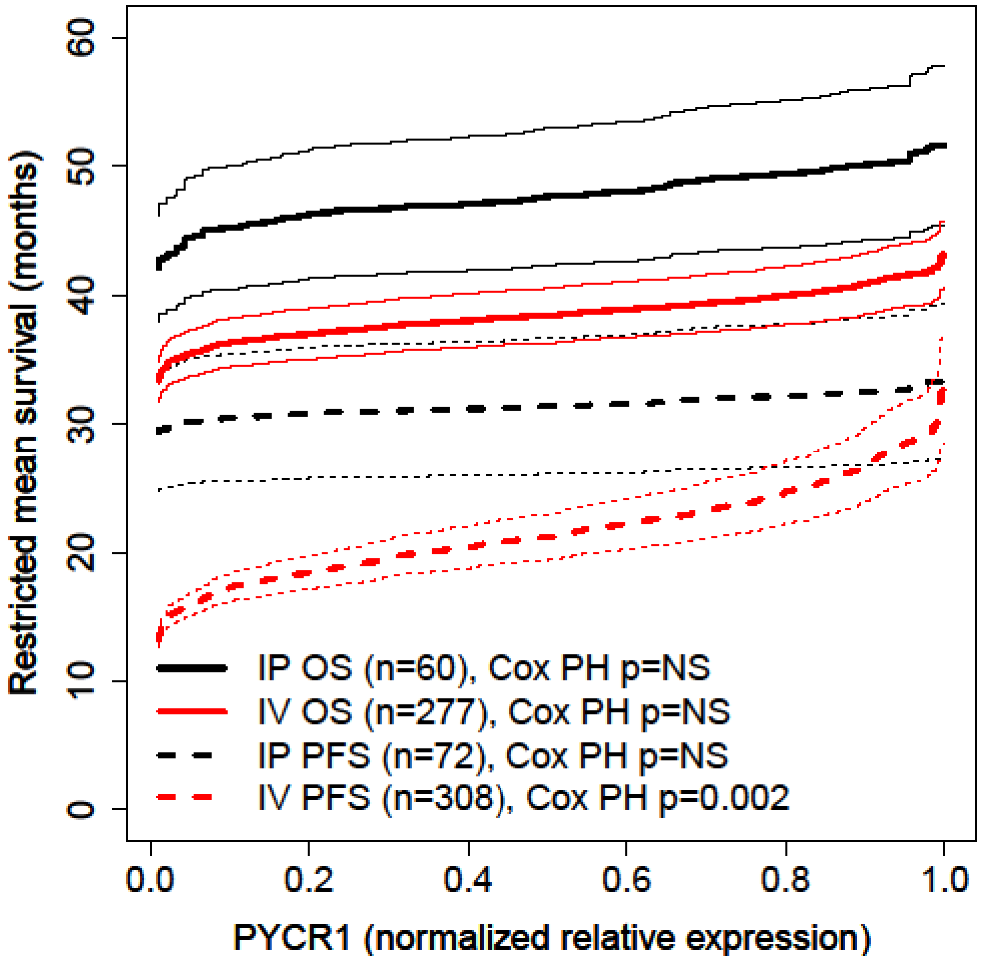
Restricted mean survival (months)
0
10
20
30
40
50
60
0.0
0.2
0.4
0.6
0.8
1.0
PYCR1 (normalized relative expression)
IP OS (n=60), Cox PH p=NS
IV OS (n=277), Cox PH p=NS
IP PFS (n=72), Cox PH p=NS
IV PFS (n=308), Cox PH p=0.002

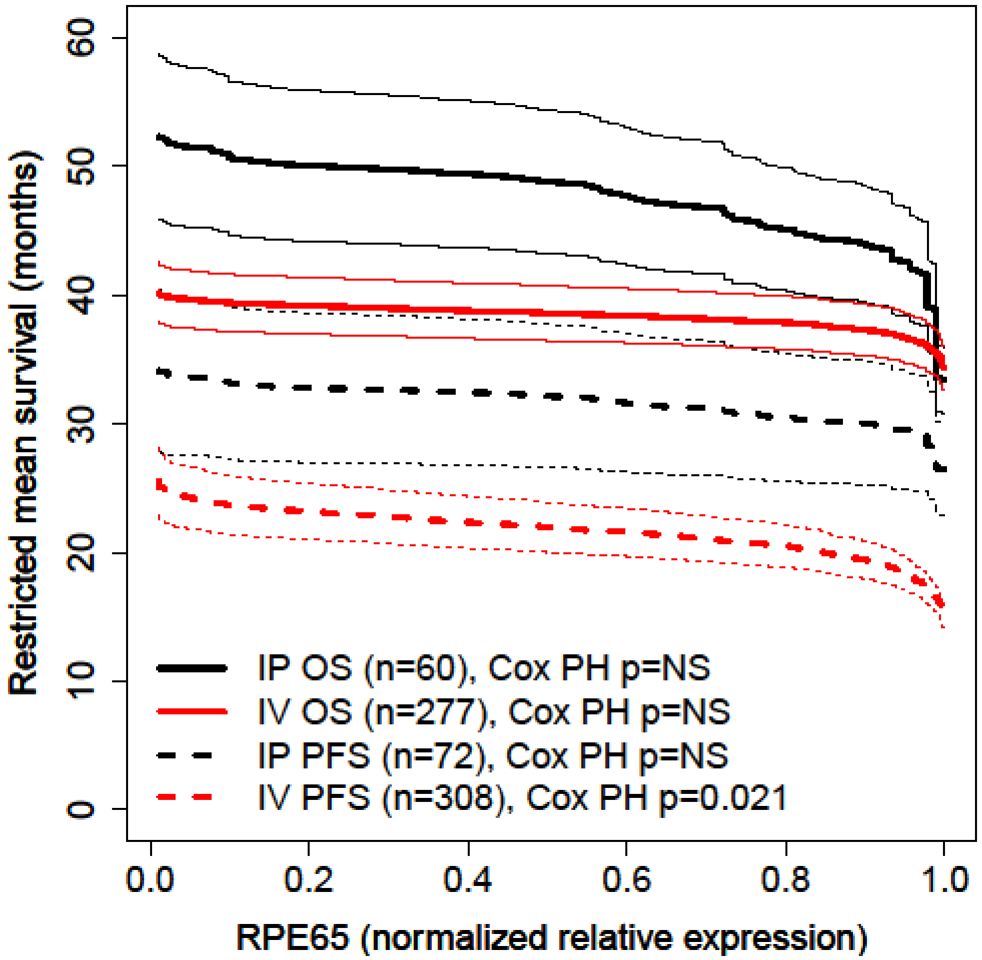
Restricted mean survival (months)
0
10
20
30
40
50
60
0.0
0.2
0.4
0.6
0.8
1.0
RPE65 (normalized relative expression)
IP OS (n=60), Cox PH p=NS
IV OS (n=277), Cox PH p=NS
IP PFS (n=72), Cox PH p=NS
IV PFS (n=308), Cox PH p=0.021

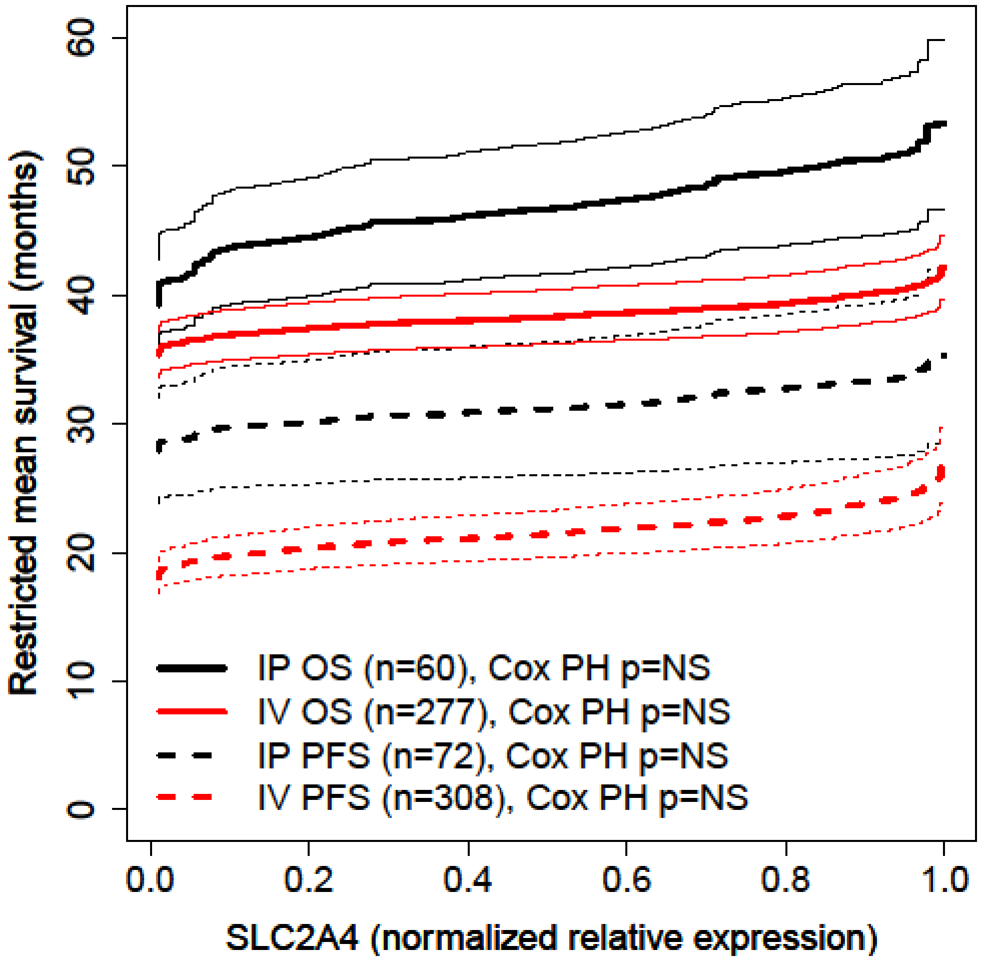

Restricted mean survival (months)
0
10
20
30
40
50
60
0.0
0.2
0.4
0.6
0.8
1.0
SLC2A4 (normalized relative expression)
IP OS (n=60), Cox PH p=NS
IV OS (n=277), Cox PH p=NS
IP PFS (n=72), Cox PH p=NS
IV PFS (n=308), Cox PH p=NS

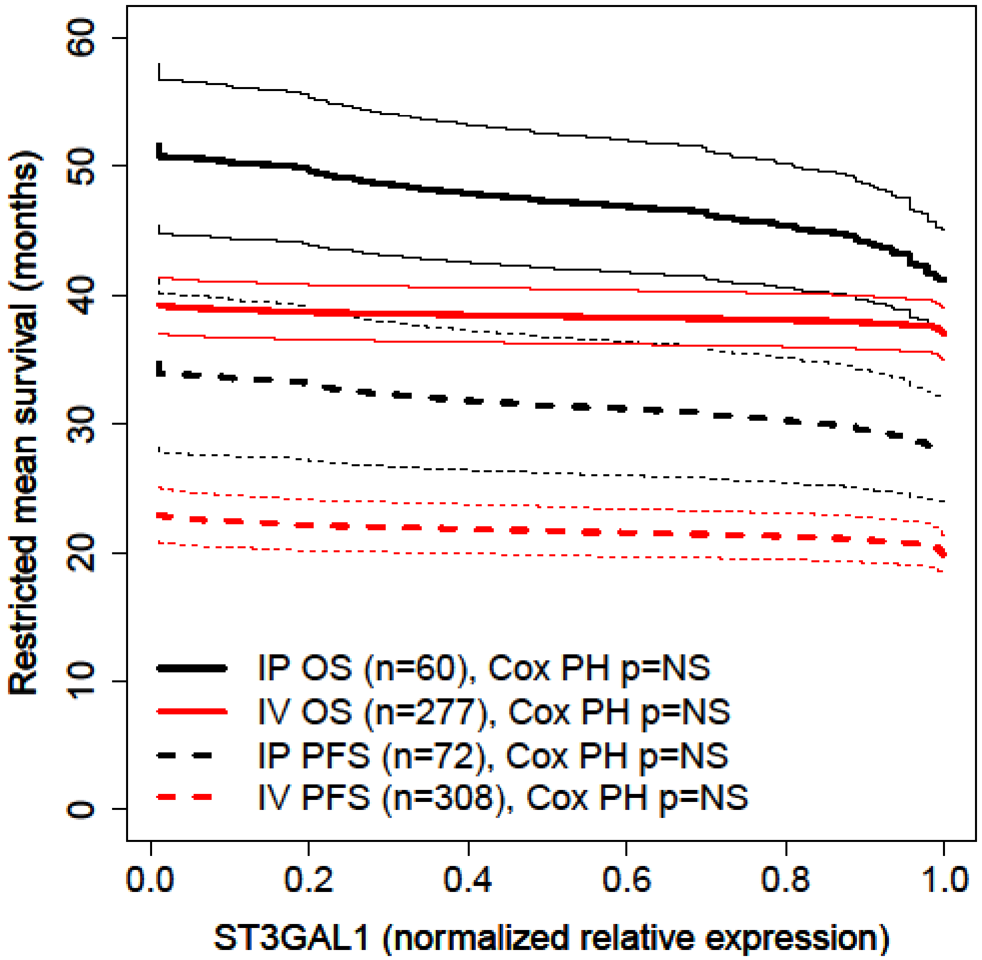

Restricted mean survival (months)
0
10
20
30
40
50
60
0.0
0.2
0.4
0.6
0.8
1.0
ST3GAL1 (normalized relative expression)
IP OS (n=60), Cox PH p=NS
IV OS (n=277), Cox PH p=NS
IP PFS (n=72), Cox PH p=NS
IV PFS (n=308), Cox PH p=NS

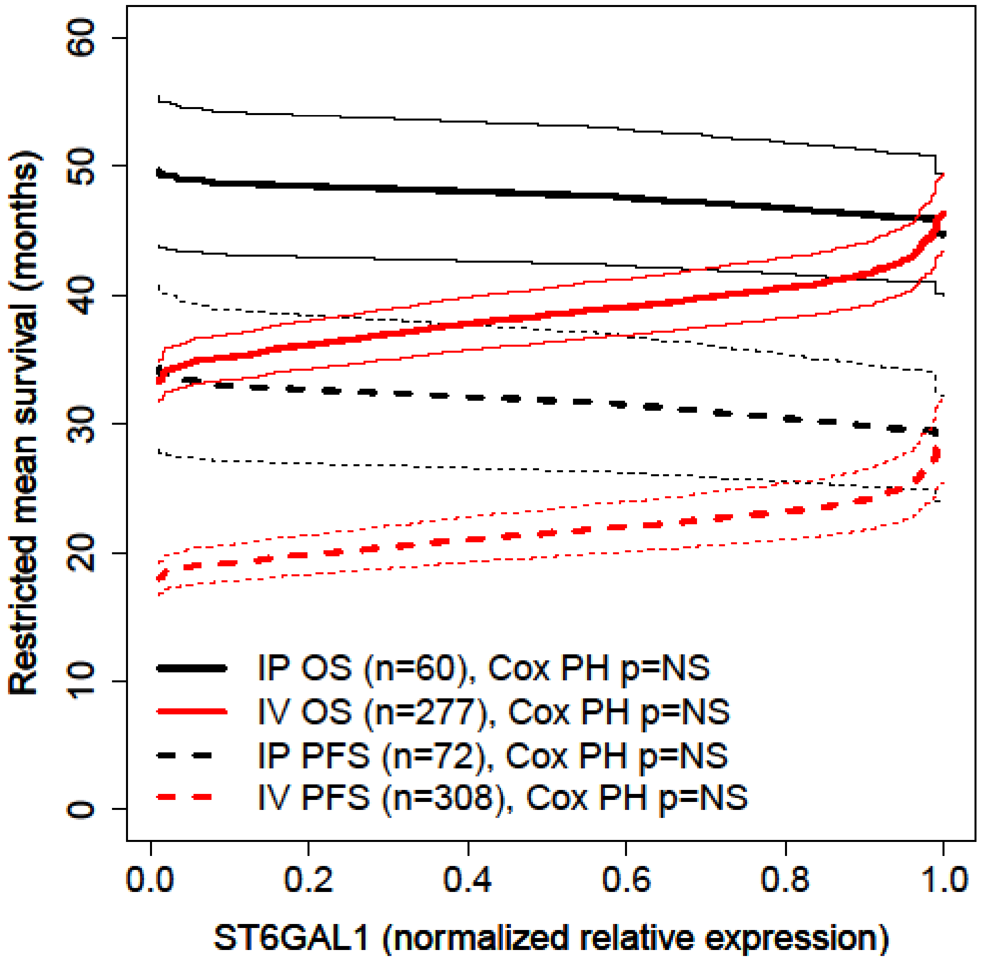

Restricted mean survival (months)
0
10
20
30
40
50
60
0.0
0.2
0.4
0.6
0.8
1.0
ST6GAL1 (normalized relative expression)
IP OS (n=60), Cox PH p=NS
IV OS (n=277), Cox PH p=NS
IP PFS (n=72), Cox PH p=NS
IV PFS (n=308), Cox PH p=NS

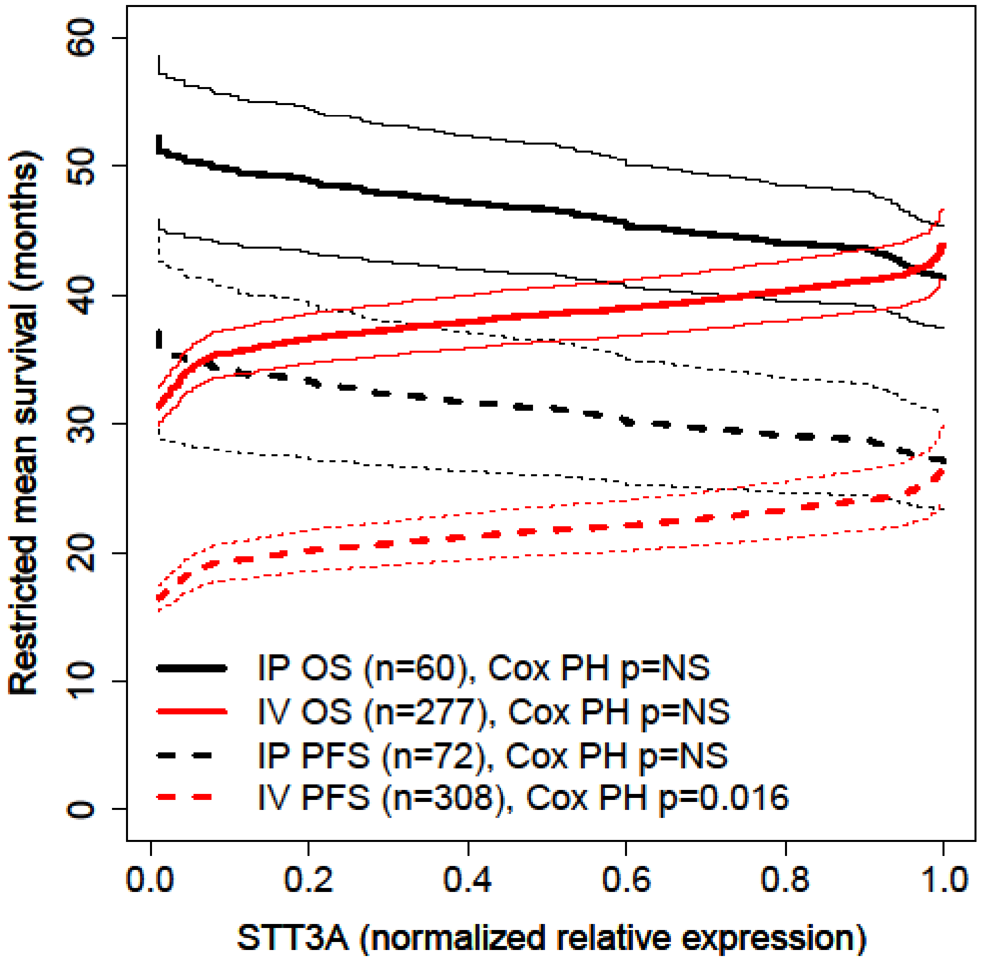

Restricted mean survival (months)
0
10
20
30
40
50
60
0.0
0.2
0.4
0.6
0.8
1.0
STT3A (normalized relative expression)
IP OS (n=60), Cox PH p=NS
IV OS (n=277), Cox PH p=NS
IP PFS (n=72), Cox PH p=NS
IV PFS (n=308), Cox PH p=0.016

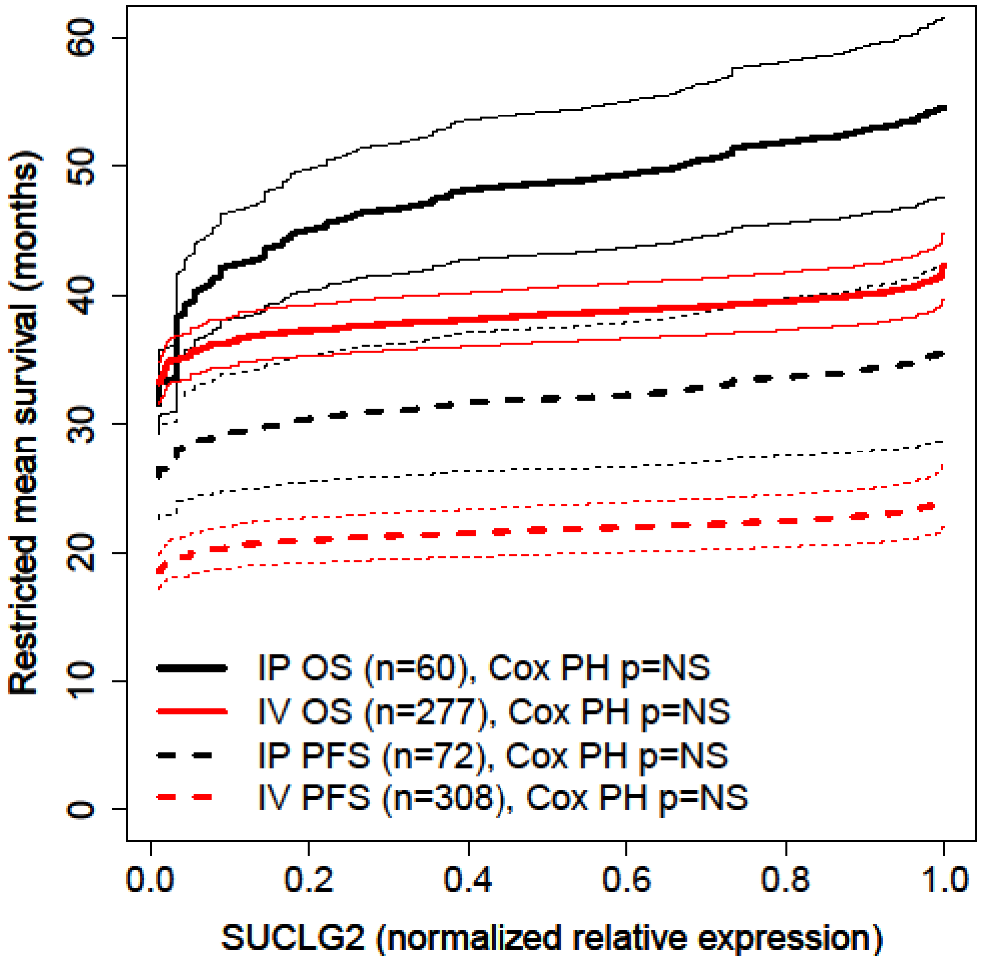

Restricted mean survival (months)
0
10
20
30
40
50
60
0.0
0.2
0.4
0.6
0.8
1.0
SUCLG2 (normalized relative expression)
IP OS (n=60), Cox PH p=NS
IV OS (n=277), Cox PH p=NS
IP PFS (n=72), Cox PH p=NS
IV PFS (n=308), Cox PH p=NS

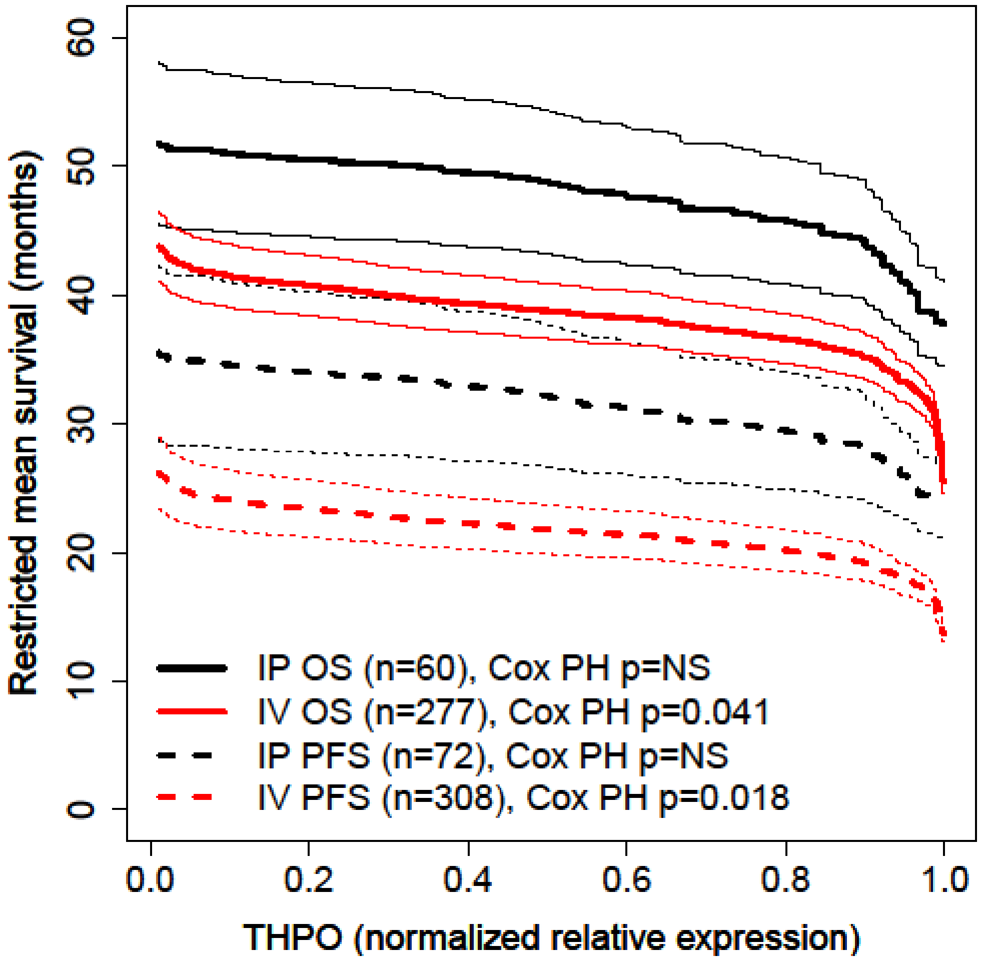
Restricted mean survival (months)
0
10
20
30
40
50
60
0.0
0.2
0.4
0.6
0.8
1.0
THPO (normalized relative expression)
IP OS (n=60), Cox PH p=NS
IV OS (n=277), Cox PH p=0.041
IP PFS (n=72), Cox PH p=NS
IV PFS (n=308), Cox PH p=0.018

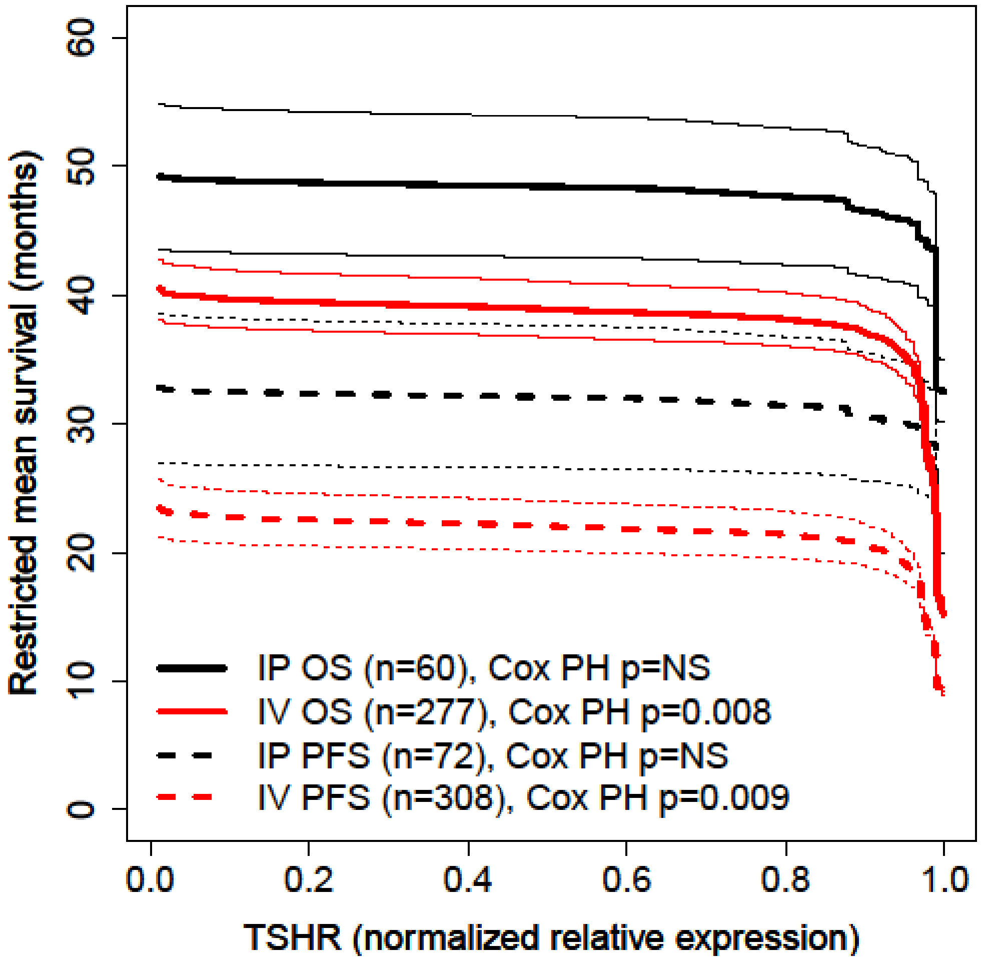

Restricted mean survival (months)
0
10
20
30
40
50
60
IP OS (n=60), Cox PH p=NS
IV OS (n=277), Cox PH p=0.008
IP PFS (n=72), Cox PH p=NS
IV PFS (n=308), Cox PH p=0.009
0.0
0.2
0.4
0.6
0.8
1.0
TSHR (normalized relative expression)

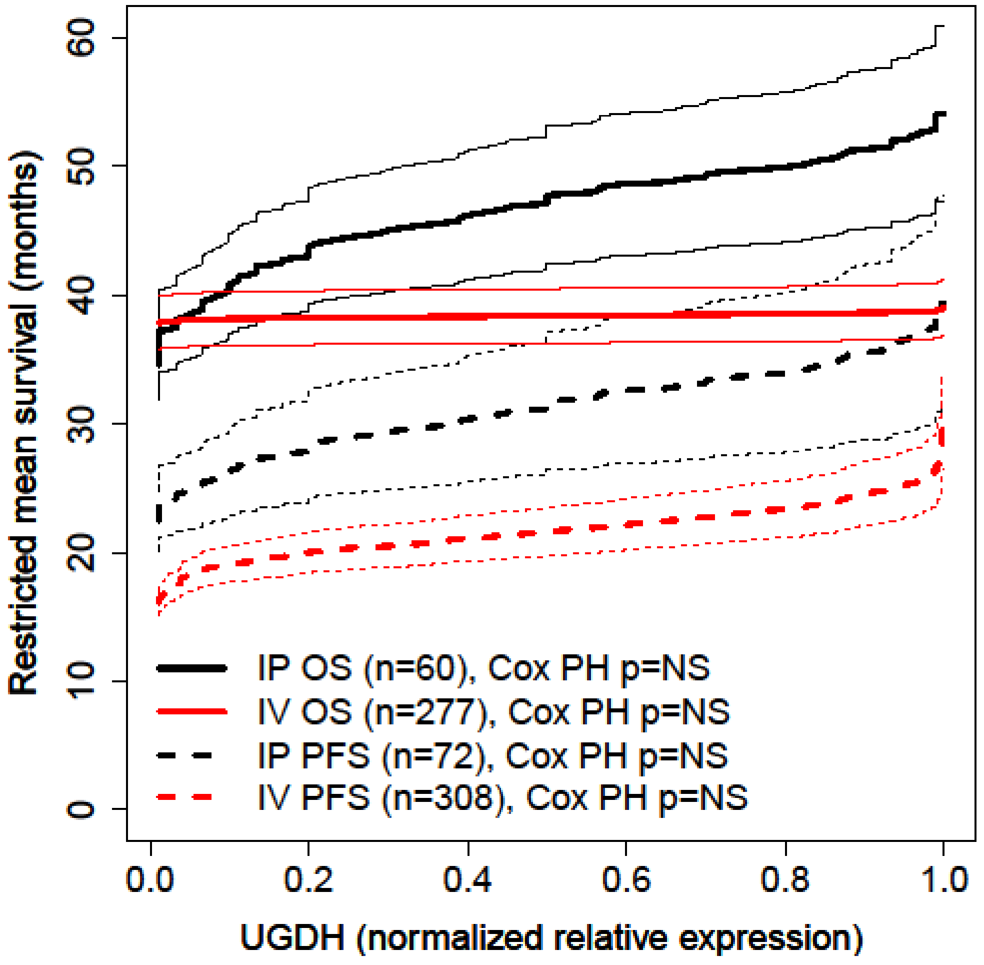
Restricted mean survival (months)
0
10
20
30
40
50
60
0.0
0.2
0.4
0.6
0.8
1.0
UGDH (normalized relative expression)
IP OS (n=60), Cox PH p=NS
IV OS (n=277), Cox PH p=NS
IP PFS (n=72), Cox PH p=NS
IV PFS (n=308), Cox PH p=NS

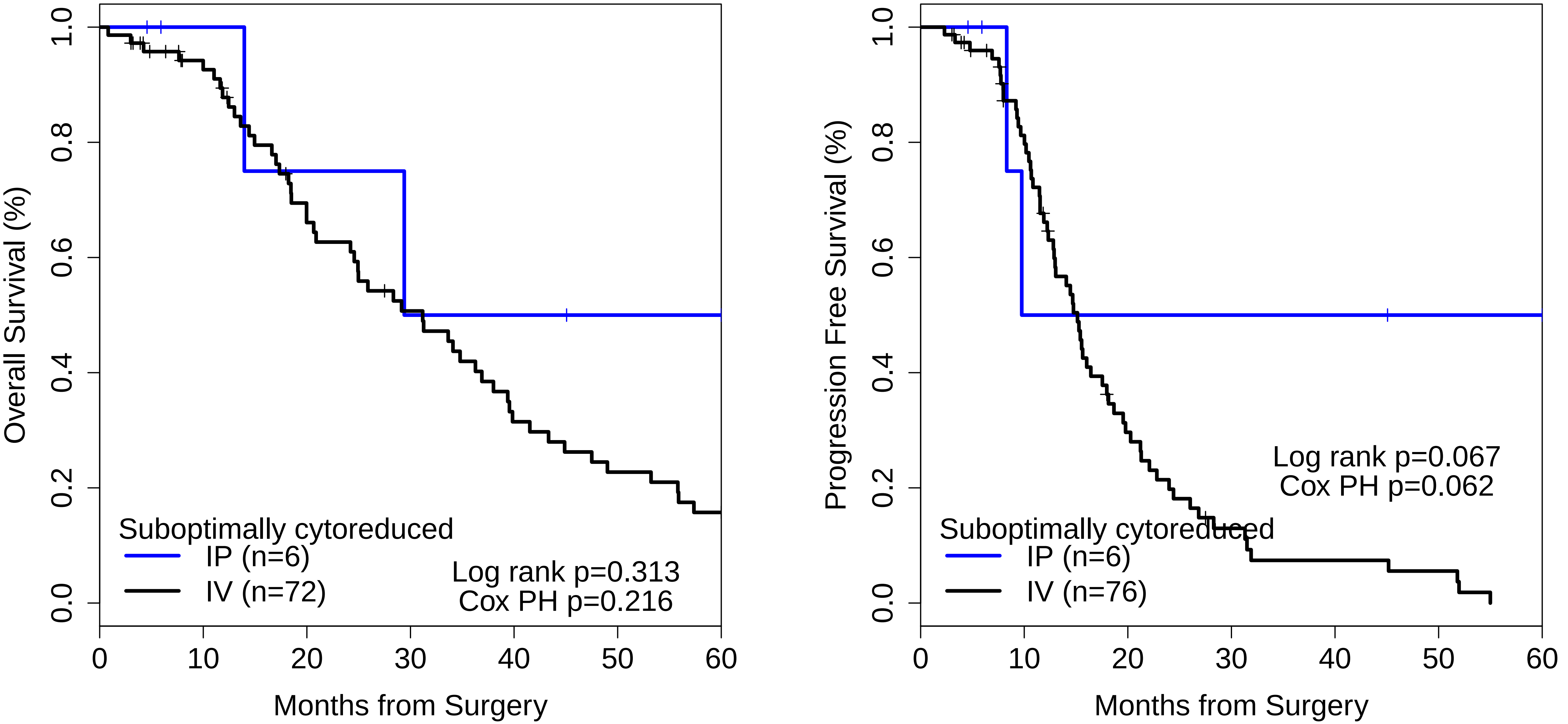

Overall Survival (%)
1.0
0.8
0.6
0.4
0.2
0.0
0
10
20
30
40
50
60
Months from Surgery
Suboptimally cytoreduced
IP (n=6)
IV (n=72)
Log rank p=0.313
Cox PH p=0.216
Progression Free Survival (%)
1.0
0.8
0.6
0.4
0.2
0.0
0
10
20
30
40
50
60
Months from Surgery
Log rank p=0.067
Cox PH p=0.062
Suboptimally cytoreduced
IP (n=6)
IV (n=76)

Supplementary Table 1. Diagram of exploratory gene analysis and validation analyses performed by this study.

<table>
<tr><td colspan="3">Number of patients selected by route of adjuvant chemotherapy administration with available clinical data</td><td>IP/IV (IP): 90
IV-only (IV): 398</td></tr>
<tr><td colspan="3">Number of patients with microarray mRNA expression data</td><td>IP: 90
IV: 396</td></tr>
<tr><td colspan="3">Number of patients with RNA-Seq mRNA expression data</td><td>IP: 34
IV: 187</td></tr>
<tr><td colspan="2">12042 genes on the mRNA microarray</td><td colspan="2">The microarray rather than the RNA-Seq expression data was used for exploratory gene analysis given that many more tumors had available microarray expression data.</td></tr>
<tr><td colspan="2">↓</td><td colspan="2">Cases divided by chemotherapy group.</td></tr>
<tr><td>IP (N=90)</td><td>IV (N=398)</td><td colspan="2">The IP and IV groups were analyzed separately.</td></tr>
<tr><td>↓</td><td>↓</td><td colspan="2">Microarray mRNA expression compared for genes from each of 229 different KEGG pathways represented on the microarray with multiple comparison p-value adjustment (FDR).</td></tr>
<tr><td>3 genes</td><td>36 genes</td><td colspan="2">Number of genes with adjusted p-values (FDR) < 0.05.
*Discovery of genes in IP group limited by low case numbers in the IP group.</td></tr>
<tr><td>↓</td><td>↓</td><td colspan="2">Validation 1: Multivariate Cox regression with microarray mRNA expression data.</td></tr>
<tr><td>0 genes</td><td>19 genes</td><td colspan="2">Number of genes significantly ($p < 0.05$) associated with OS and/or PFS from multivariate regression, using only significant (likelihood ratio $p < 0.05$) regression models.</td></tr>
<tr><td>↓</td><td>↓</td><td colspan="2">Validation 2: Multivariate Cox regression with RNA-Seq mRNA expression data.</td></tr>
<tr><td>0 genes</td><td>3 genes</td><td colspan="2">Number of genes significantly ($p < 0.05$) associated with OS and PFS from multivariate regression, using only significant (likelihood ratio $p < 0.05$) regression models.</td></tr>
</table>

Supplementary Table 2. Adjuvant chemotherapy groups compared by progression free survival times < 12 months (Early) versus ≥ 12 months (Late)

	IP Early (n=9)	IP Late (n=45)	p-value	IV Early (n=106)	IV Late (n=202)	p-value
Mean Age	58.9	53.1	0.133[a]	60.8	59.0	0.181[a]
Stage			0.244[b]			0.002[b]
IIA/B/C	1	0		1	18	
IIIA/B	0	2		1	16	
IIIC	8	29		75	117	
IV	0	4		17	25	
Grade			0.935[b]			0.339[b]
G2	1	4		11	28	
G3	8	29		82	145	
Residual Disease			0.486[b]			0.234[b]
> 20 mm	1	2		15	35	
11-20 mm	1	2		8	9	
1-10 mm	5	18		55	68	
None	1	8		11	45	
Adjuvant Chemotherapy Outcome			0.003[c]			5.905×10^{-12}[c]
Complete Response	4	31		40	142	
Partial Response	2	0		27	11	
Progressive Disease	1	0		16	4	
Stable Disease	1	1		5	6	
Platinum Status			7.63×10^{-8}[c]			$< 2.2 \times 10^{-16}$[c]
Resistant	8	0		73	7	
Sensitive	1	33		14	143	

[a]t-test. [b]Kruskal-Wallis test. [c]Fisher's Exact test.
Numbers shown for each comparison do not add to the total number of patients in each adjuvant chemotherapy group due to incomplete data reporting or inadequate follow up time. Missing data is omitted from statistical comparisons. IP: Intraperitoneal. IV: Intravenous.

Supplementary Table 3. Associations of differentially expressed gene tumor microarray mRNA expression levels with survival outcomes (univariate analysis).

Group	Gene	p-value[a]	Fold change[b]	Overall survival HR (95% CI)[c], p-value	Progression free survival HR (95% CI)[c], p-value
IV	AASS	0.010	0.922	1.31 (0.90-1.91), 0.164	**1.53 (1.12-2.10), 0.008**
IV	ALPI	0.043	0.972	2.40 (0.77-7.45), 0.131	2.01 (0.76-5.35), 0.162
IV	APC2	0.003	0.956	**3.90 (1.34-11.38), 0.013**	**3.74 (1.52-9.21), 0.004**
IV	ASPA	0.010	0.950	**3.61 (1.71-7.63), < 0.001**	**2.34 (1.17-4.68), 0.016**
IV	BCAT1	0.006	1.621	0.92 (0.85-1.01), 0.076	**0.88 (0.81-0.95), 0.001**
IV	CD82	0.040	1.190	0.97 (0.77-1.24), 0.824	0.83 (0.67-1.02), 0.076
IV	CHST1	0.025	1.318	0.88 (0.77-1.02), 0.086	0.99 (0.89-1.11), 0.886
IV	CMA1	0.018	0.961	1.55 (0.65-3.70), 0.326	1.85 (0.80-4.31), 0.151
IV	DGAT1	0.016	1.254	1.06 (0.88-1.26), 0.551	0.90 (0.77-1.04), 0.161
IV	FBXO2	0.050	0.769	1.12 (0.98-1.29), .0103	1.12 (0.99-1.25), 0.062
IV	FUT8	0.031	1.243	0.90 (0.78-1.03), 0.122	0.94 (0.82-1.06), 0.303
IV	FUT9	0.049	0.971	1.27 (.49-3.31), 0.620	**4.02 (1.74-9.28), 0.001**
IV	FZD4	0.027	1.291	**0.82 (0.71-0.96), 0.014**	**0.83 (0.72-0.95), 0.009**
IV	FZD5	0.027	1.285	**0.72 (0.62-0.85), < 0.001**	**0.73 (0.63-0.84), < 0.001**
IV	GCNT1	0.001	1.189	0.77 (0.59-1.02), 0.064	**0.67 (0.52-0.85), < 0.001**
IV	GCNT4	0.046	0.967	2.63 (0.92-7.53), 0.071	**2.75 (1.14-6.62), 0.024**
IV	GPAA1	0.003	1.258	0.90 (0.73-1.11), 0.317	0.87 (0.73-1.02), 0.092
IV	HLCS	0.034	1.043	0.52 (0.26-1.04), 0.065	0.57 (0.32-1.03), 0.061
IV	LMAN2	0.031	1.172	0.93 (0.71-1.22), 0.618	0.84 (0.67-1.05), 0.120
IV	MTHFR	0.001	0.953	**5.30 (1.91-14.70), 0.001**	**2.59 (1.12-6.01), 0.027**
IV	NANS	0.006	1.222	0.91 (0.73-1.14), 0.407	**0.80 (0.65-0.97), 0.027**
IV	NCAM2	0.041	0.960	**3.81 (1.54-9.41), 0.004**	**2.78 (1.24-6.23), 0.013**
IV	NRG1	0.023	0.956	1.32 (0.58-2.97), 0.510	**2.74 (1.19-6.29), 0.018**
IV	PAH	0.007	0.956	**3.32 (1.44-7.66), 0.005**	2.03 (0.96-4.30), 0.064
IV	PER1	0.003	0.877	**1.68 (1.22-2.33), 0.002**	**1.63 (1.21-2.19), 0.001**
IV	PLA2G2F	0.019	0.962	1.64 (0.62-4.28), 0.317	1.39 (.59-3.25), 0.452
IV	PYCR1	0.046	1.170	0.80 (0.63-1.02), 0.071	**0.65 (0.53-0.81), < 0.001**
IV	RPE65	0.016	0.971	1.90 (0.51-7.10), 0.343	3.25 (0.97-10.95), 0.057
IV	SLC2A4	0.047	1.041	0.56 (0.22-1.43), 0.224	0.51 (0.23-1.14), 0.102
IV	ST3GAL1	0.046	1.250	1.03 (0.88-1.21), 0.741	1.04 (0.91-1.18), 0.603
IV	ST6GAL1	0.022	1.123	**0.65 (0.46-0.91), 0.013**	**0.74 (0.56-0.99), 0.042**
IV	STT3A	0.017	1.220	**0.78 (0.63-0.97), 0.025**	**0.83 (0.69-0.98), 0.032**
IV	SUCLG2	0.027	1.165	0.83 (0.64-1.07), 0.145	0.89 (0.72-1.11), 0.299
IV	THPO	0.002	0.967	**7.25 (1.73-30.43), 0.007**	**4.00 (1.10-14.59), 0.036**
IV	TSHR	0.018	0.879	**1.59 (1.20-2.09), 0.001**	**1.43 (1.14-1.78), 0.002**
IV	UGDH	0.029	1.196	0.98 (0.81-1.19), 0.851	**0.82 (0.69-0.98), 0.031**
IP	DAO	0.043	1.125	0.86 (0.04-20.72), 0.926	0.59 (0.05-6.94), 0.671
IP	FBP2	0.022	1.124	**0.04 (0.00-0.67), 0.025**	0.15 (0.02-1.52), 0.110
IP	GPI	0.025	0.607	0.85 (0.46-1.55), 0.588	1.48 (0.82-2.65), 0.190

[a]Adjusted p-values for differentially expressed genes within each adjuvant chemotherapy group after stratification by progression free survival (PFS) < versus ≥ 12 months.

[b]Fold change indicates relative level of tumor gene expression among patients with PFS ≥ 12 months compared to patients with PFS < 12 months.

[c]HR (95% CI): Hazard ratio (95% Confidence Interval), p-value calculated by univariate Cox proportional hazards regression. Significant associations are in bold type for convenient reference.

Supplementary Table 4. Associations of differentially expressed gene tumor microarray mRNA expression levels with survival outcomes (multivariate analysis).

Group	Gene	p-value[a]	Fold change[b]	Overall survival HR (95% CI)[c], p-value	Progression free survival HR (95% CI)[c], p-value
IV	AASS	0.010	0.922	0.98 (0.65-1.50), 0.945	1.31 (0.92-1.86), 0.134
IV	ALPI	0.043	0.972	2.32 (0.73-7.38), 0.153	2.47 (0.92-6.59), 0.072
IV	APC2	0.003	0.956	**4.62 (1.46-14.59), 0.009**	**6.35 (2.42-16.69), < 0.001**
IV	ASPA	0.010	0.950	**4.24 (1.92-9.36), < 0.001**	**2.59 (1.24-5.38), 0.011**
IV	BCAT1	0.006	1.621	**0.90 (0.82-0.98), 0.019**	**0.85 (0.78-0.92), < 0.001**
IV	CD82	0.040	1.190	1.11 (0.85-1.46), 0.436	0.90 (0.71-1.15), 0.407
IV	CHST1	0.025	1.318	0.92 (0.78-1.07), 0.274	1.03 (0.91-1.18), 0.621
IV	CMA1	0.018	0.961	1.49 (0.62-3.59), 0.370	**2.66 (1.13-6.21), 0.024**
IV	DGAT1	0.016	1.254	1.07 (0.88-1.30), 0.500	0.86 (0.72-1.02), 0.074
IV	FBXO2	0.050	0.769	1.04 (0.89-1.22), 0.592	1.06 (0.93-1.21), 0.403
IV	FUT8	0.031	1.243	0.92 (0.79-1.07), 0.279	0.89 (0.77-1.03), 0.125
IV	FUT9	0.049	0.971	1.77 (.064-4.88), 0.271	**6.23 (2.61-14.86), < 0.001**
IV	FZD4	0.027	1.291	**0.84 (0.72-1.00), 0.044**	**0.78 (0.67-0.92), 0.002**
IV	FZD5	0.027	1.285	**0.72 (0.61-0.86), < 0.001**	**0.71 (0.61-0.83), < 0.001**
IV	GCNT1	0.001	1.189	0.81 (0.60-1.10), 0.173	**0.65 (0.50-0.84), 0.001**
IV	GCNT4	0.046	0.967	2.20 (0.72-6.72), 0.167	**2.67 (1.01-7.01), 0.047**
IV	GPAA1	0.003	1.258	0.94 (0.75-1.18), 0.581	0.84 (0.69-1.01), 0.062
IV	HLCS	0.034	1.043	**0.39 (0.19-0.79), 0.010**	**0.39 (0.20-0.76), 0.006**
IV	LMAN2	0.031	1.172	0.94 (0.71-1.25), 0.662	0.82 (0.64-1.04), 0.100
IV	MTHFR	0.001	0.953	2.93 (0.98-8.81), 0.055	1.59 (0.64-3.98), 0.317
IV	NANS	0.006	1.222	1.01 (0.79-1.28), 0.948	**0.76 (0.61-0.94), 0.012**
IV	NCAM2	0.041	0.960	**3.11 (1.19-8.08), 0.020**	**2.85 (1.20-6.79), 0.018**
IV	NRG1	0.023	0.956	0.79 (0.31-2.01), 0.613	2.44 (0.93-6.38), 0.069
IV	PAH	0.007	0.956	**3.30 (1.44-7.54), 0.005**	1.89 (0.85-4.21), 0.118
IV	PER1	0.003	0.877	**1.52 (1.07-2.16), 0.020**	**1.52 (1.11-2.10), 0.010**
IV	PLA2G2F	0.019	0.962	1.70 (0.58-4.95), 0.337	1.82 (0.71-4.70), 0.216
IV	PYCR1	0.046	1.170	0.96 (0.75-1.23), 0.749	**0.71 (0.57-0.89), 0.002**
IV	RPE65	0.016	0.971	1.51 (0.37-6.18), 0.565	**4.28 (1.25-14.70), 0.021**
IV	SLC2A4	0.047	1.041	0.70 (0.26-1.93), 0.493	0.48 (0.20-1.13), 0.092
IV	ST3GAL1	0.046	1.250	1.11 (0.92-1.34), 0.289	1.00 (0.86-1.17), 0.973
IV	ST6GAL1	0.022	1.123	0.72 (0.52-1.02), 0.064	0.79 (0.59-1.06), 0.118
IV	STT3A	0.017	1.220	0.80 (0.64-1.01), 0.066	**0.79 (0.65-0.96), 0.016**
IV	SUCLG2	0.027	1.165	0.97 (0.74-1.29), 0.853	0.96 (0.76-1.22), 0.764
IV	THPO	0.002	0.967	**4.71 (1.07-20.73), 0.041**	**5.34 (1.34-21.29), 0.018**
IV	TSHR	0.018	0.879	**1.47 (1.10-1.96), 0.008**	**1.38 (1.08-1.77), 0.009**
IV	UGDH	0.029	1.196	1.07 (0.87-1.31), 0.523	0.86 (0.71-1.03), 0.094
IP	DAO	0.043	1.125	0.11 (0.00-17.22), 0.387	0.18 (0.00-3.98), 0.280
IP	FBP2	0.022	1.124	0.00 (0.00-0.22), 0.012[d]	0.04 (0.00-0.80), 0.035[d]
IP	GPI	0.025	0.607	0.93 (0.42-2.06), 0.854	1.53 (0.82-2.86), 0.180

[a]Adjusted p-values for differentially expressed genes within each adjuvant chemotherapy group after stratification by progression free survival (PFS) < versus ≥ 12 months.

[b]Fold change indicates relative level of tumor gene expression among patients with PFS ≥ 12 months compared to patients with PFS < 12 months.

[c]HR (95% CI): Hazard ratio (95% Confidence Interval), p-value calculated by Cox proportional hazards regression adjusted for covariates age, surgical stage and histologic grade. Significant associations are in bold type for convenient reference.

[d]Not significant for gene listed because the likelihood ratio test p-value for the overall Cox proportional hazards regression model is not significant (p > 0.05).

Supplementary Table 5. Associations of differentially expressed gene tumor RNA-Seq mRNA expression levels with survival outcomes.

Group	Gene	RNA-Seq Normalized expression values	
		Overall survival<br>HR (95% CI)[a], p-value	Progression free survival<br>HR (95% CI)[a], p-value
IV	AASS	0.98 (0.82-1.17), 0.823	**1.18 (1.00-1.38), 0.044**
IV	ALPI	1.14 (0.76-1.72), 0.531	1.20 (0.91-1.60), 0.201
IV	APC2	0.91 (0.74-1.12), 0.360	1.01 (0.88-1.16), 0.897
IV	ASPA	1.03 (0.86-1.24), 0.736	1.06 (0.87-1.29), 0.560
IV	BCAT1	0.95 (0.80-1.14), 0.591	1.02 (0.82-1.25), 0.893
IV	CD82	1.19 (0.96-1.47), 0.119	1.02 (0.84-1.23), 0.873
IV	CHST1	0.85 (0.64-1.14), 0.287	0.97 (0.81-1.17), 0.739
IV	CMA1	1.07 (0.92-1.24), 0.379	1.11 (0.96-1.30), 0.139
IV	DGAT1	1.16 (0.95-1.43), 0.148	0.97 (0.78-1.21), 0.787
IV	FBXO2	0.98 (0.79-1.22), 0.888	0.97 (0.84-1.13), 0.727
IV	FUT8	0.81 (0.63-1.04), 0.095	0.99 (0.82-1.21), 0.954
IV	FUT9	0.99 (0.81-1.22), 0.949	1.11 (0.92-1.32), 0.269
IV	FZD4	0.85 (0.66-1.09), 0.188	0.82 (0.65-1.03), 0.087
IV	FZD5	0.91 (0.69-1.20), 0.513	0.94 (0.72-1.21), 0.618
IV	GCNT1	**0.76 (0.59-0.97), 0.031**	**0.79 (0.66-0.95), 0.011**
IV	GCNT4	0.52 (0.23-1.15), 0.104	0.80 (0.64-1.01), 0.056
IV	GPAA1	1.10 (0.88-1.38), 0.394	0.96 (0.79-1.17), 0.695
IV	HLCS	0.85 (0.70-1.03), 0.098	0.98 (0.83-1.16), 0.843
IV	LMAN2	1.03 (0.83-1.26), 0.820	0.93 (0.78-1.10), 0.398
IV	MTHFR	0.99 (0.79-1.24), 0.939	1.17 (0.98-1.39), 0.088
IV	NANS	1.04 (0.86-1.25), 0.722	0.87 (0.73-1.04), 0.120
IV	NCAM2	**1.21 (1.02-1.42), 0.025**	**1.23 (1.05-1.45), 0.011**
IV	NRG1	0.91 (0.76-1.10), 0.332	1.20 (1.00-1.44), 0.056
IV	PAH	1.08 (0.88-1.32), 0.473	1.09 (0.90-1.32), 0.383
IV	PER1	1.01 (0.78-1.29), 0.955	1.15 (0.94-1.41), 0.170
IV	PLA2G2F	**1.34 (1.10-1.64), 0.004**	**1.64 (1.04-2.60), 0.033**
IV	PYCR1	1.04 (0.85-1.29), 0.685	0.88 (0.73-1.07), 0.196
IV	RPE65	0.95 (0.47-1.94), 0.890	1.21 (0.77-1.90), 0.411
IV	SLC2A4	1.01 (0.83-1.24), 0.894	1.06 (0.85-1.32), 0.619
IV	ST3GAL1	1.18 (0.95-1.48), 0.143	1.16 (0.93-1.44), 0.187
IV	ST6GAL1	0.80 (0.64-1.01), 0.062	0.85 (0.71-1.01), 0.063
IV	STT3A	0.92 (0.74-1.13), 0.432	0.84 (0.70-1.01), 0.067
IV	SUCLG2	0.98 (0.81-1.20), 0.861	0.99 (0.82-1.18), 0.890
IV	THPO	1.11 (0.80-1.55), 0.533	0.84 (0.66-1.05), 0.121
IV	TSHR	**1.71 (1.31-2.22), < 0.001**	**1.64 (1.22-2.21), 0.001**
IV	UGDH	0.88 (0.73-1.07), 0.198	0.82 (0.67-1.01), 0.066
IP	DAO	7.62 (0.29-197.84), 0.222	1.11 (0.26-4.74), 0.889
IP	FBP2	0.07 (0.00-9.75), 0.293[b]	5.27 (0.23-120.00), 0.298
IP	GPI	0.10 (0.00-3.44), 0.203	1.94 (1.02-3.67), 0.042[c]

[a]HR (95% CI): Hazard ratio (95% Confidence Interval), p-value calculated by Cox proportional hazards regression adjusted for covariates age and surgical stage. HRs are adjusted to 1 standard deviation of gene expression for each gene to aid between-gene interpretations regarding the association of gene expression with survival. For instance, a HR of 1.30 for gene X would indicate that the risk of death increased 30% for each 1 standard deviation increase in mRNA expression of gene X across the range of observed expression values of gene X. Significant associations are in bold type for convenient reference.

[b]Stage was omitted from this regression model to prevent a failure to converge error.

[c]Not significant for gene listed because the likelihood ratio test p-value for the overall Cox proportional hazards regression model is not significant ($p > 0.05$).
